# Supplementary material for: Hist2Cell: Deciphering fine-grained cellular architectures from histology images
Source: Cell Genom. 2026 Jan 26;6(3):101137. doi: 10.1016/j.xgen.2025.101137 (PMC12985364; doi:10.1016/j.xgen.2025.101137)
Supplement: Document S1. Figures S1–S7 and Tables S1–S3 [file mmc1.pdf]

Cell Genomics, Volume *6*

Supplemental information

Hist2Cell: Deciphering fine-grained cellular architectures from histology images

Weiqin Zhao, Zhuo Liang, Xianjie Huang, Yuanhua Huang, and Lequan Yu

SUPPLEMENTAL INFORMATION

SUPPLEMENTAL FIGURES

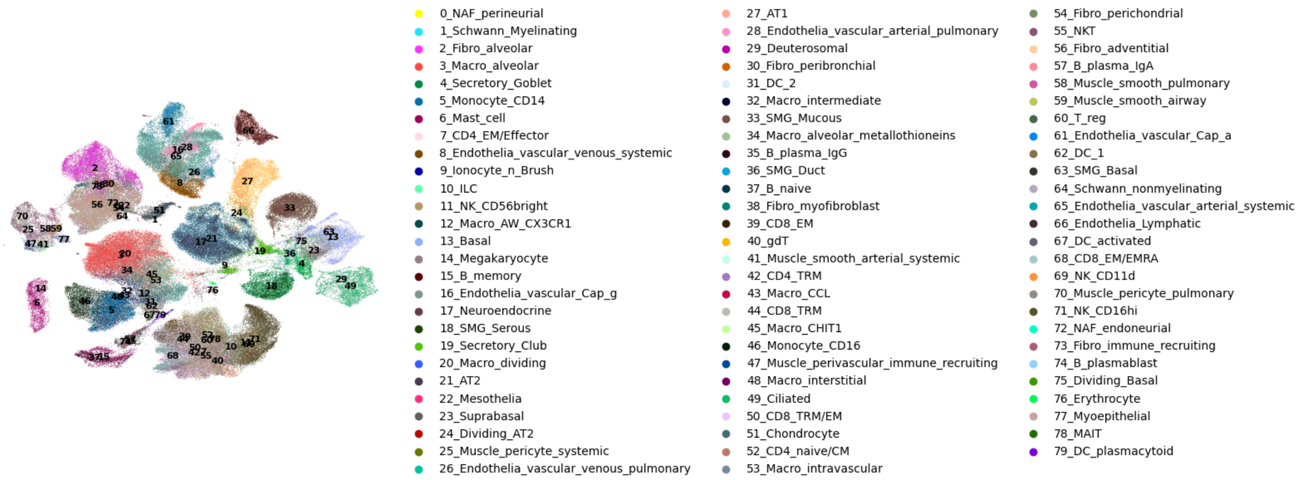

Figure S1. UMAP of the human lung reference, related to Figure 2
UMAP representation of 80 cell types identified by Louvain clustering on the reference single cell reference dataset of human lung dataset.

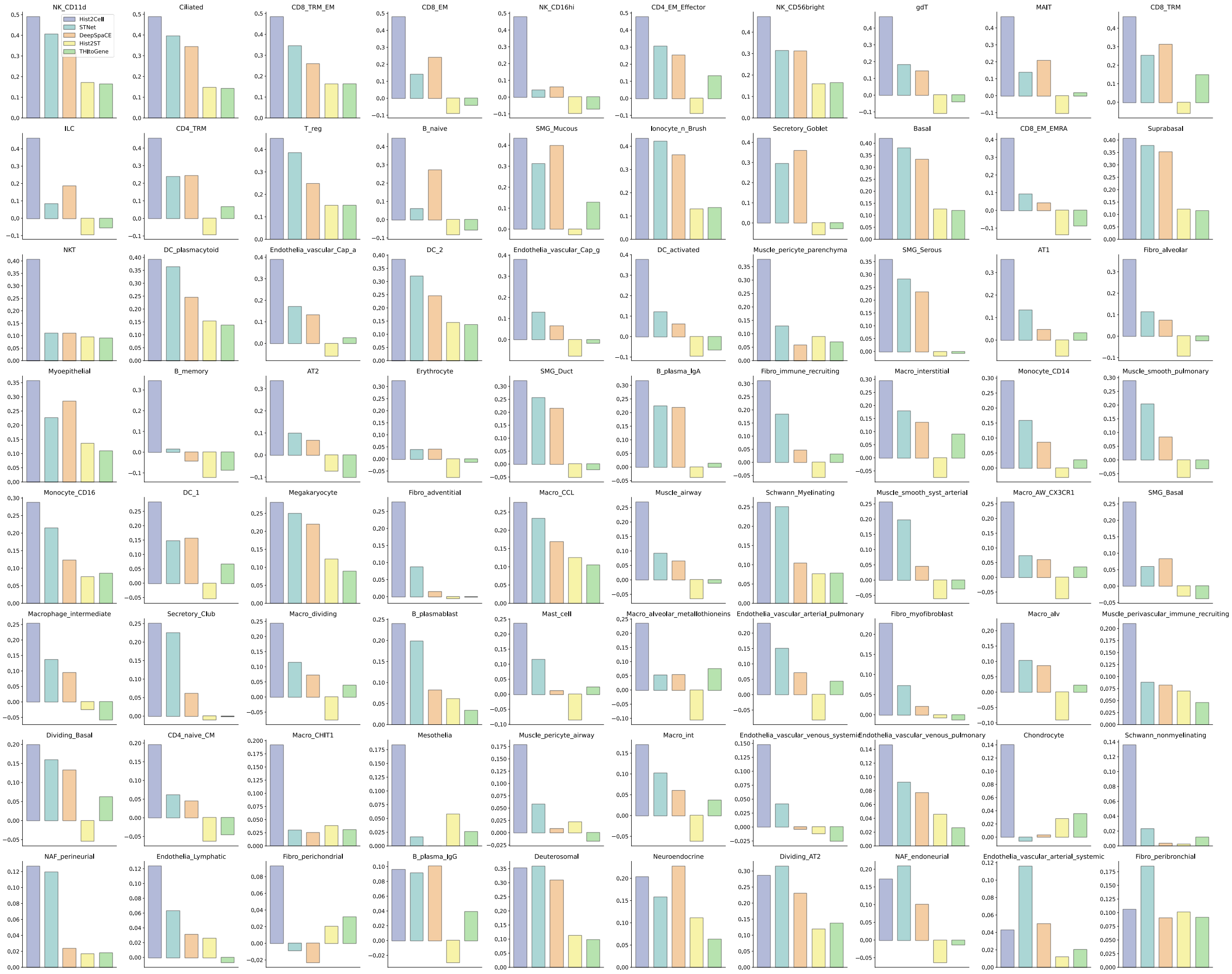

Figure S2. Average Pearson's R comparison of all methods on the human lung dataset, related to Figure 2

Histogram representing the average Pearson's R values for all methods of all cell types on the human lung dataset.

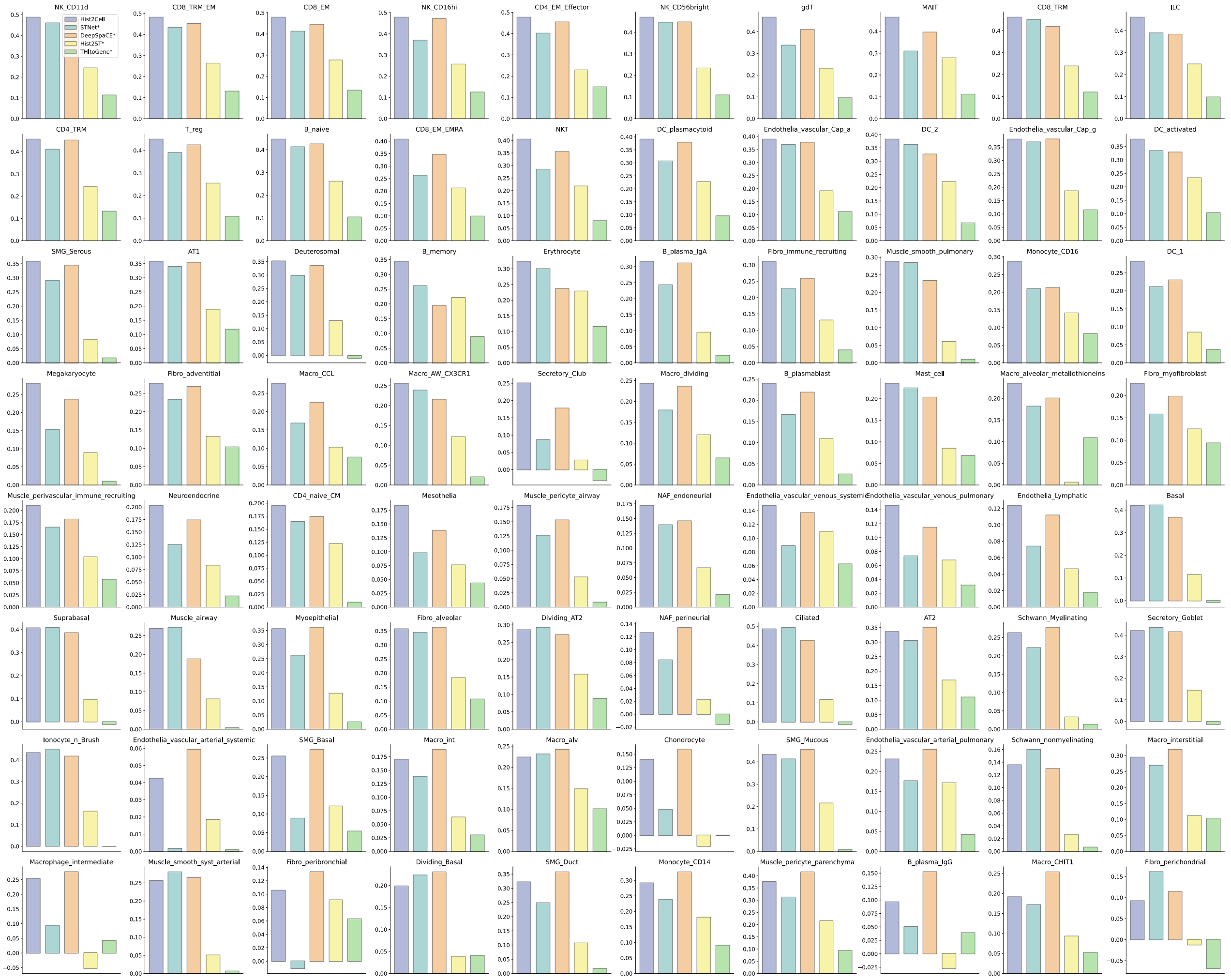

Figure S3. Performance comparison of adapted baseline methods and Hist2Cell on the human lung dataset, related to Figure 2

Histogram representing the average Pearson's R values for all adapted (*) baseline methods and Hist2Cell of all cell types on the human lung dataset.

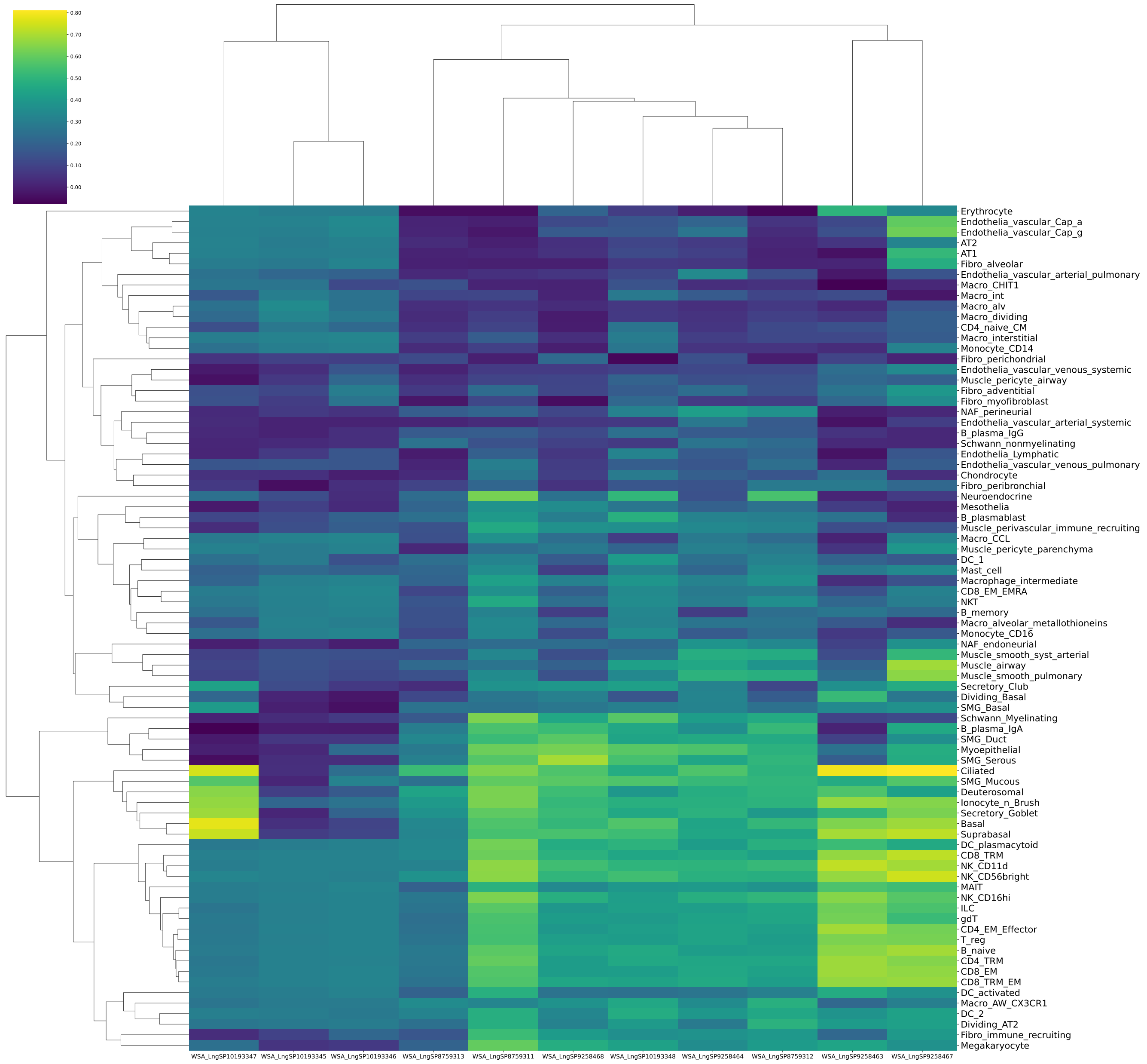

Figure S4. Pearson R Clustermap of Hist2Cell performance on the human lung dataset, related to Figure 2

Clustermap colored by the Pearson R from Hist2Cell for different cell types (y-axis) on different slides (x-axis) during testing in the human lung dataset experiments. Color intensity represents the value of Pearson R. The top 30% cell types across 11 slides have a mean Pearson R 0.50.

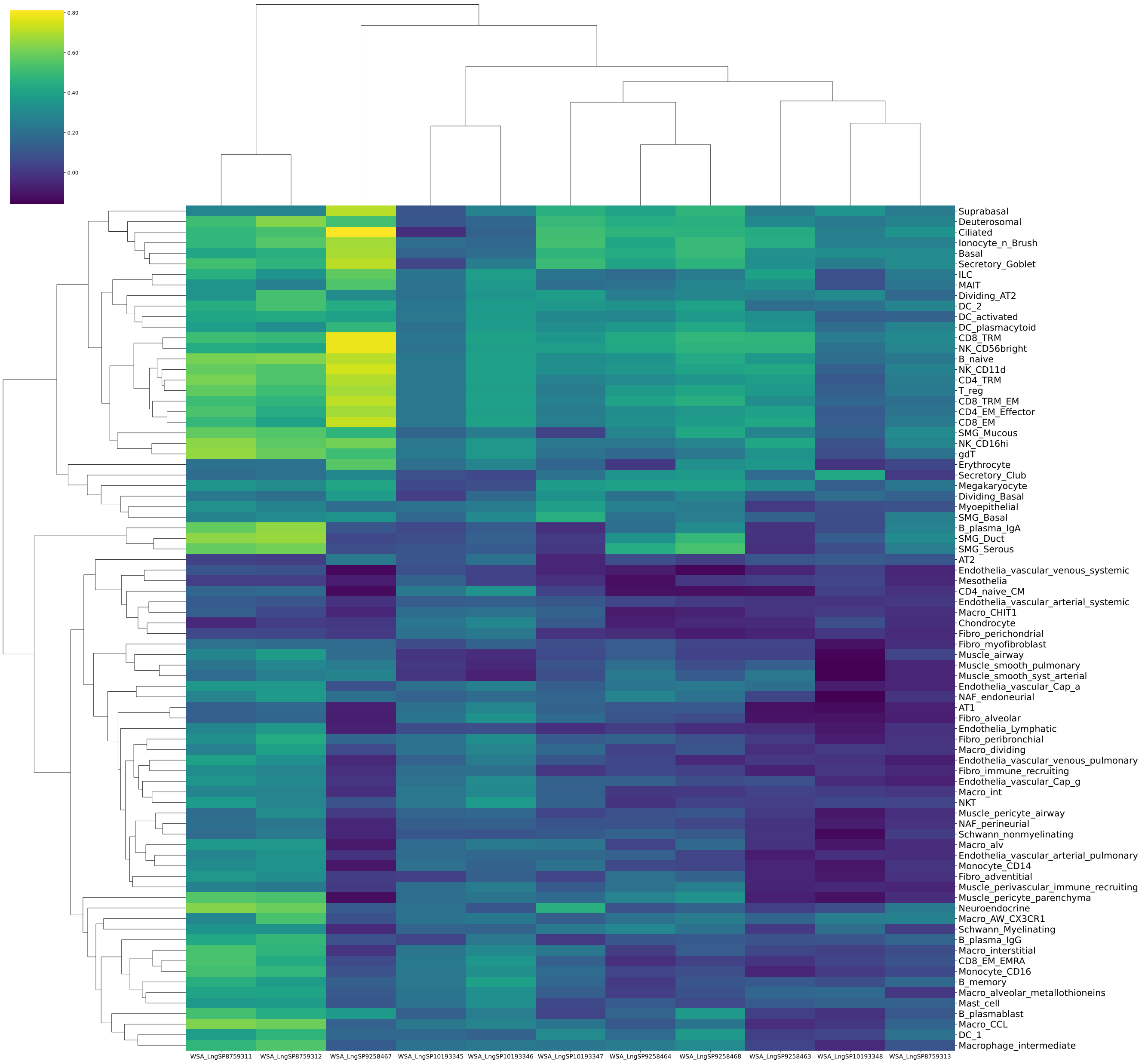

Figure S5. Pearson R Clustermap of DeepSpaCE baseline performance on the human lung dataset, related to Figure 2

Clustermap colored by the Pearson R from DeepSpaCE baseline for different cell types (y-axis) on different slides (x-axis) during testing in the human lung dataset experiments. Color intensity represents the value of Pearson R. The top 30% cell types across 11 slides have a mean Pearson R 0.43.

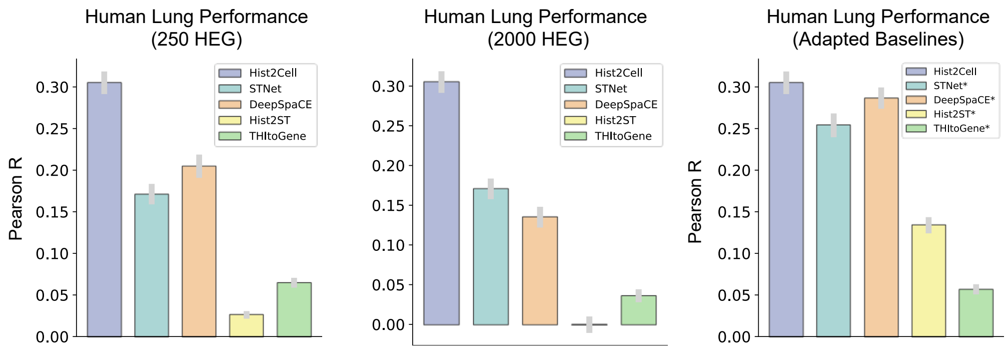

Figure S6. Performance evaluation of cell abundance prediction on the healthy human lung dataset, related to Figure 2

Histogram depicting the average Pearson's R values for cell abundance prediction in the leave-one-donor-out cross-validation experiment conducted on the healthy human lung dataset. Error bars represent standard error across the cross-validation folds. ”250 HEG” and ”2000 HEG” represent that, for the previous ST prediction baselines, we follow their setup to first predict the 250/2000 highly expressed genes, and then use the predicted highly expressed genes to estimate the cell abundance. ”Adapted Baselines” represents that, for the previous ST prediction baselines, we adapted them to our one-stage prediction strategy to directly predict the fine-grained cell abundances from the histology image. We use * to distinguish the adapted baselines from the original ones. Results show that Hist2Cell demonstrated more accurate predictions of fine-grained cell type abundances across locations, as previous ST prediction baselines suffer from low performance in fine-grained cell type abundances prediction under both ”250 HEG” and ”2000 HEG” and setups as their noisy and unstable predictions for a relatively small group of genes. When adapted, the performance of previous baselines increases obviously, showing the efficacy of our one-stage prediction strategy.

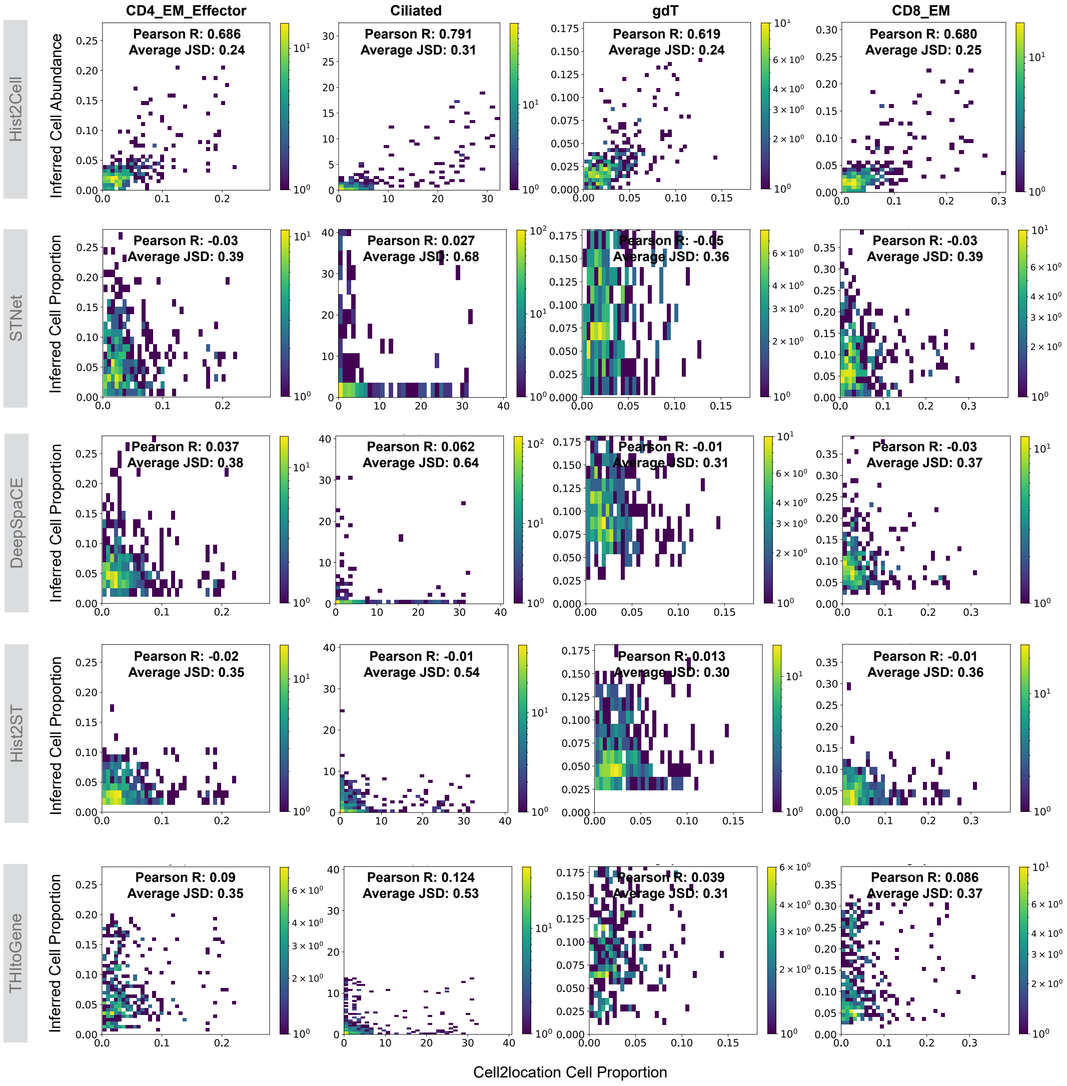

Figure S7. Concordance analysis of Hist2Cell versus baseline methods on human lung tissue, related to Figure 2

2D histogram plots showcasing the concordance of cell abundance between ground truth (x-axis) and prediction of Hist2Cell and baseline methods (y-axis) across all testing spots in the healthy human lung slide. Color denotes 2D histogram counts. Pearson's R denotes Pearson's correlation coefficient, and JSD denotes Jensen–Shannon divergence. Color intensity represents the spot frequency.

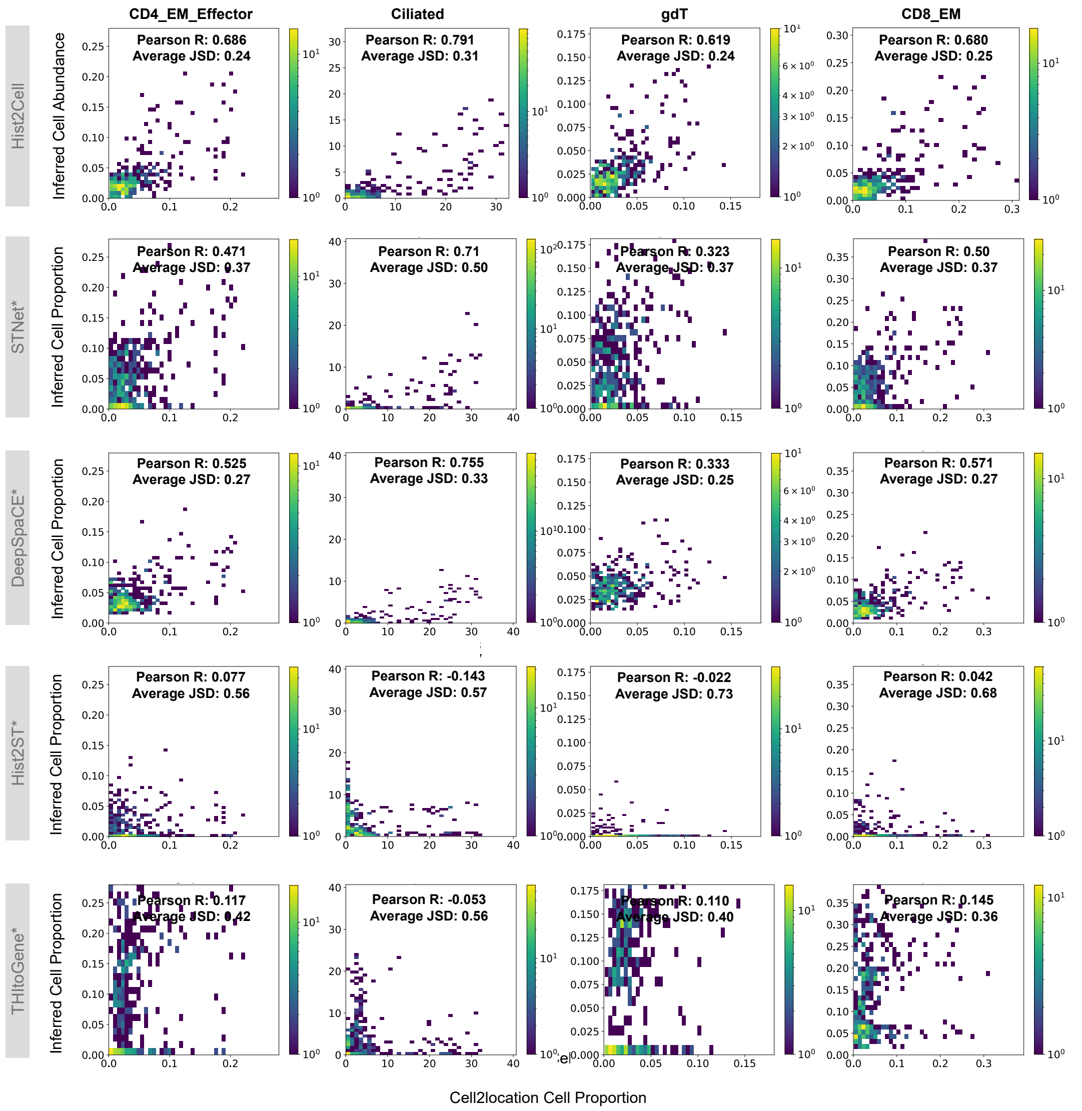

Figure S8. Concordance analysis of Hist2Cell versus adapted baseline methods on human lung tissue, related to Figure 2

2D histogram plots showcasing the concordance of cell abundance between ground truth (x-axis) and prediction of Hist2Cell and the "adapted" (*) baseline methods (y-axis) across all testing spots in the healthy human lung slide. Color denotes 2D histogram counts. Pearson's R denotes Pearson's correlation coefficient, and JSD denotes Jensen–Shannon divergence. Color intensity represents the spot frequency.

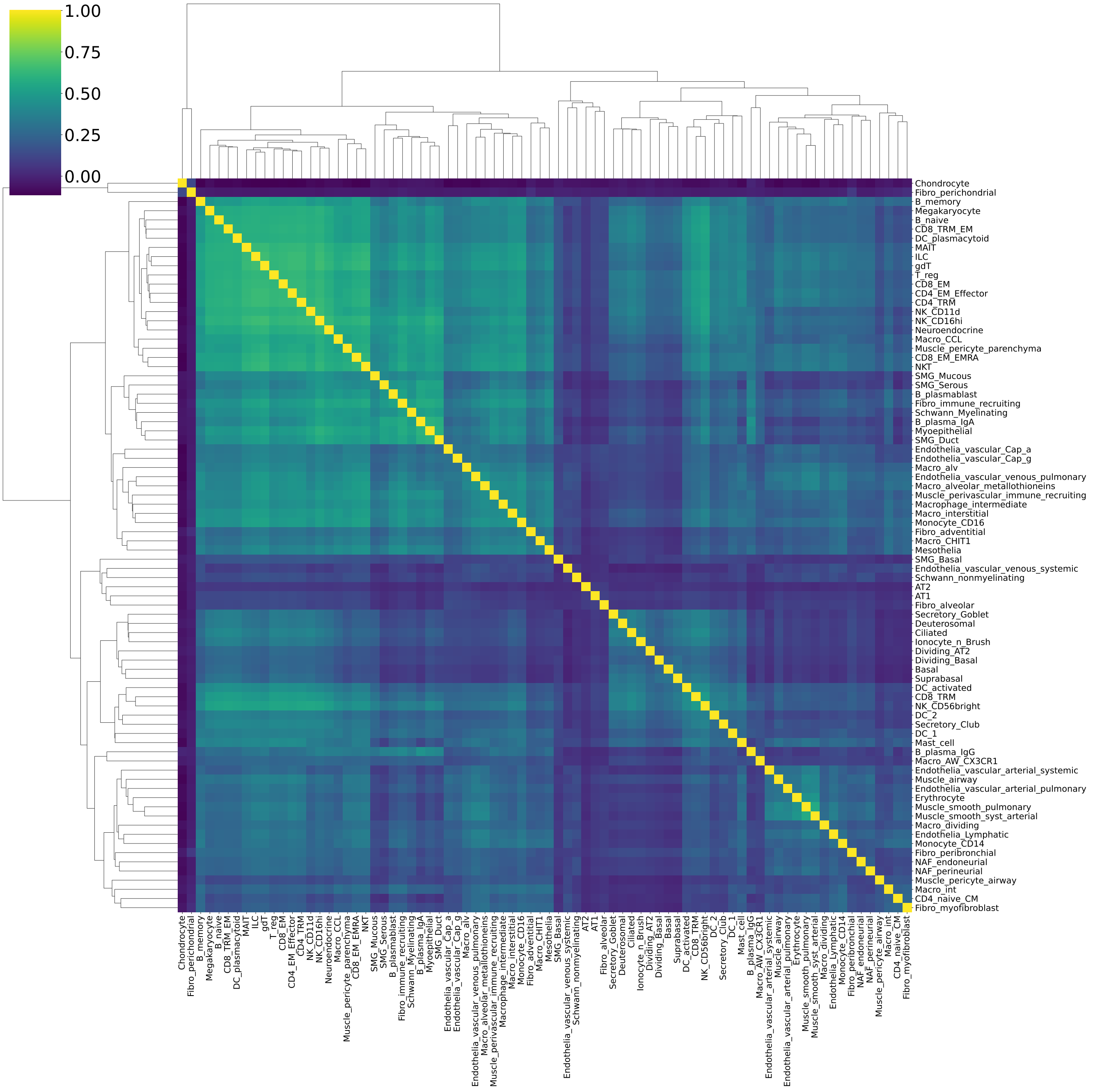

Figure S9. Detailed Moran's R Clustermaps of ground truth cell abundances in human lung tissue, related to Figure 2

Detailed Human lung Moran's R Clustermaps calculated from ground truth cell type abundances (cell2location algorithm) in Figure 2G. Color intensity represents the value of Moran's R.

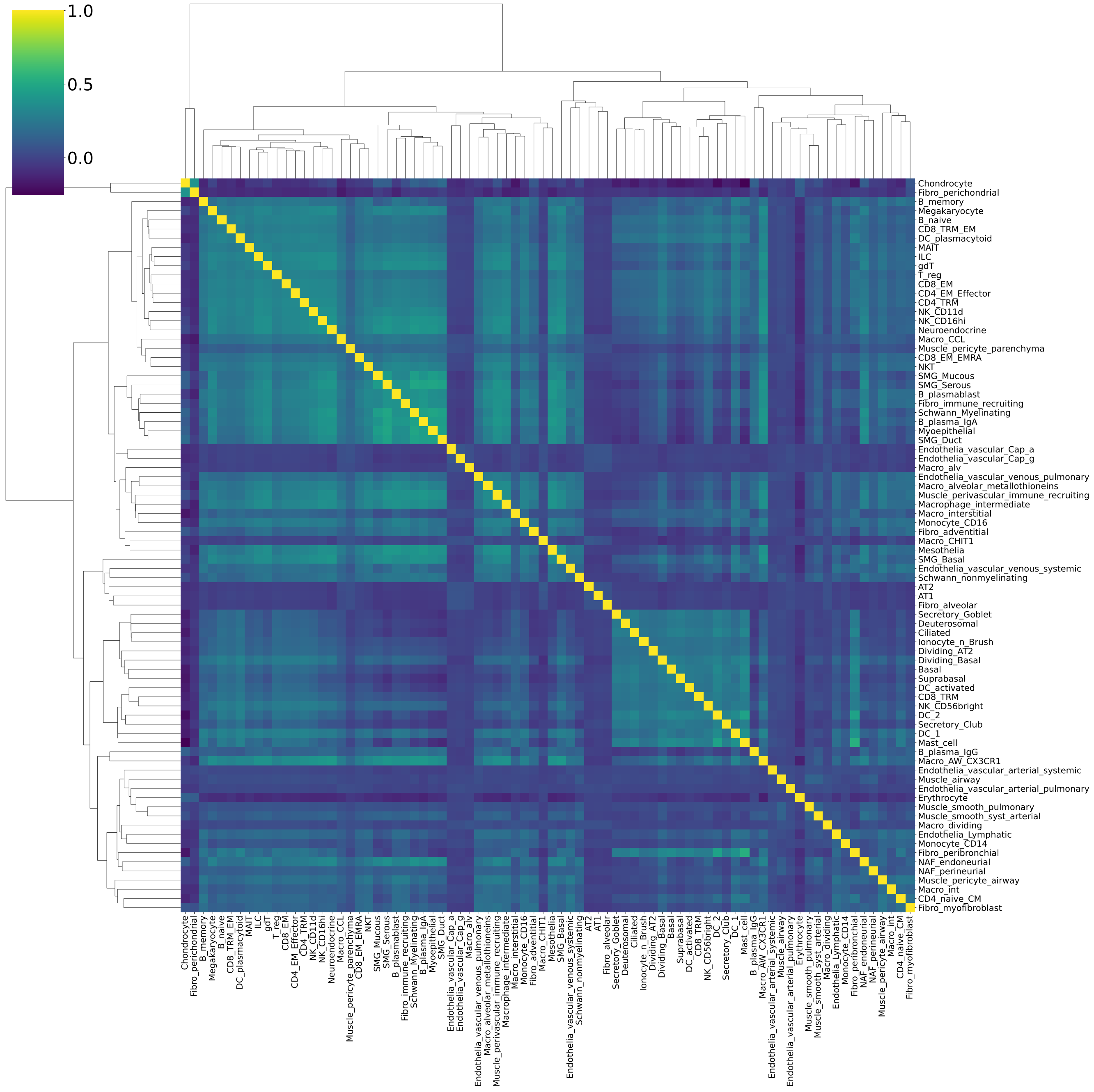

Figure S10. Detailed Moran's R Clustermaps of Hist2Cell predictions in human lung tissue, related to Figure 2

Detailed Human lung Moran's R Clustermaps calculated from Hist2Cell predictions in Figure 2G. Color intensity represents the value of Moran's R.

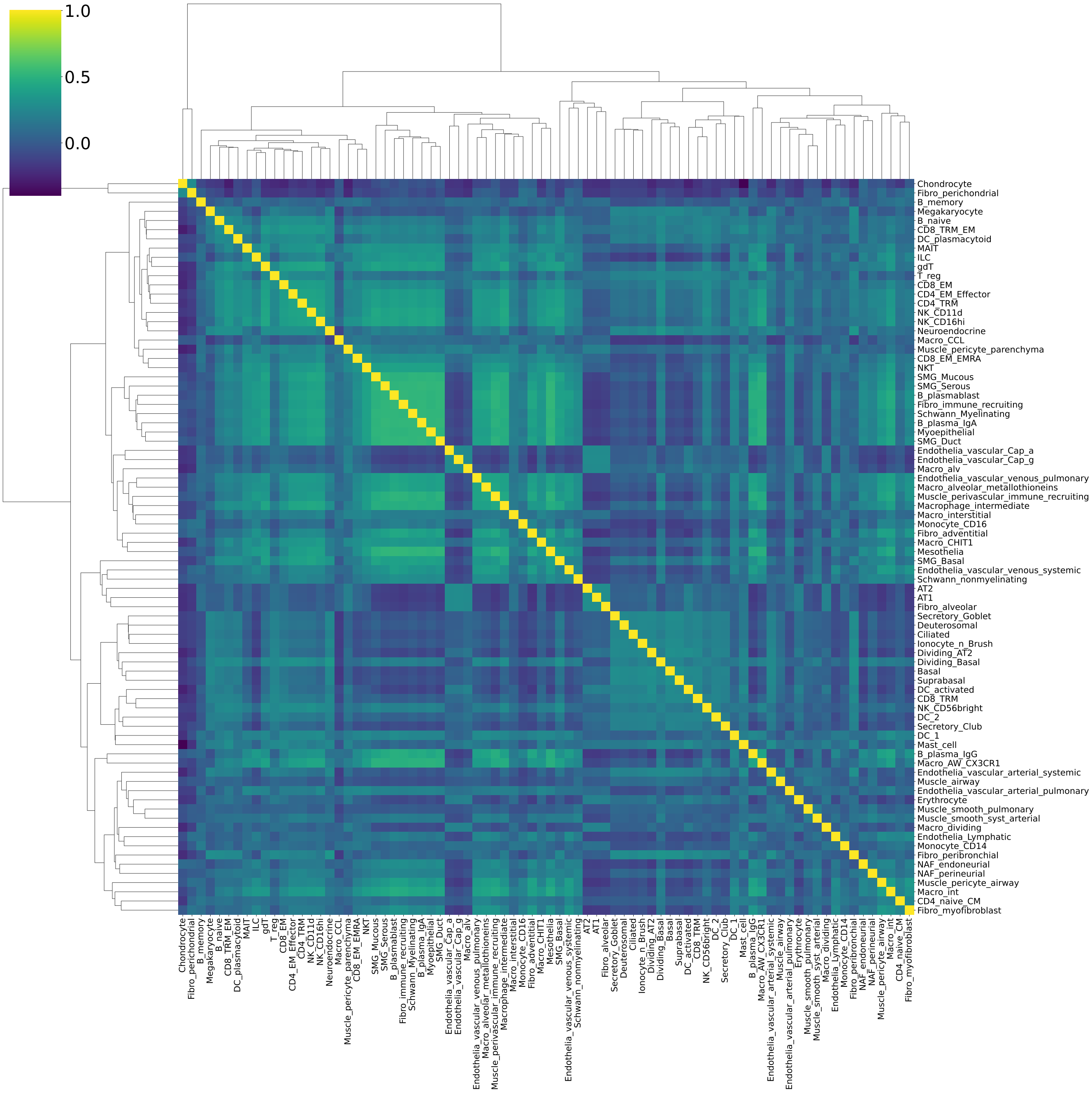

Figure S11. Detailed Moran's R Clustermaps of DeepSpaCE* predictions in human lung tissue, related to Figure 2

Detailed Human lung Moran's R Clustermaps calculated from DeepSpaCE* predictions in Figure 2G. Color intensity represents the value of Moran's R.

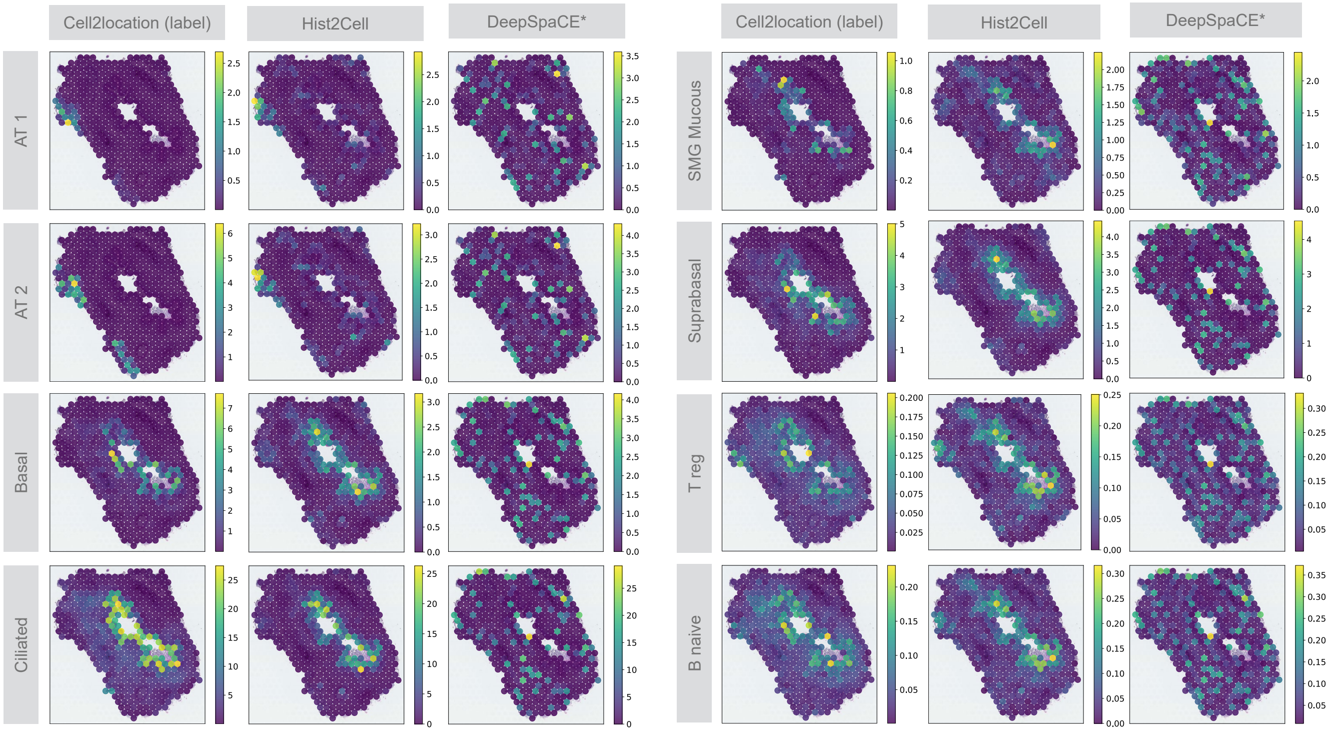

Figure S12. Hist2Cell provide more accurate alignment to manual annotation than DeepSpaCE*, related to Figure 3

The visualizations comparing the spatial cell abundances as determined by ground truth, Hist2Cell, and DeepSpaCE* predictions for select key cell types. Hist2Cell show less false positives than DeepSpaCE* when comparing to both ground truth and manual annotation in Figure 3B. Color intensity represents the value of cell abundance.

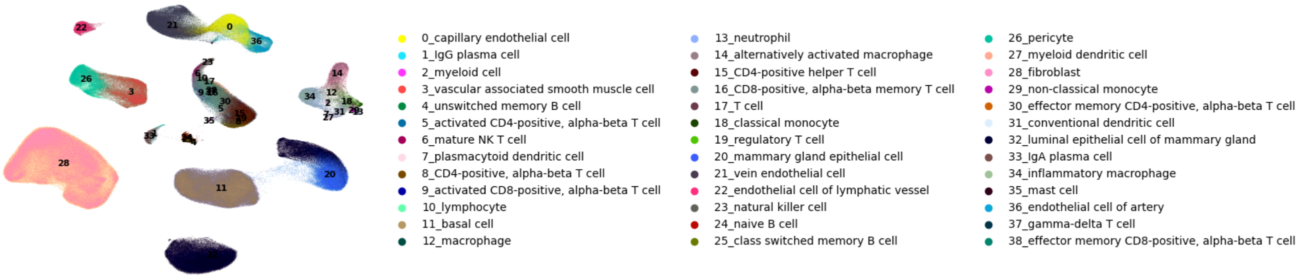

Figure S13. UMAP of the human breast cancer reference, related to Figure 4

UMAP representation of 39 cell types identified by Louvain clustering on the reference single cell reference dataset of human breast cancer dataset.

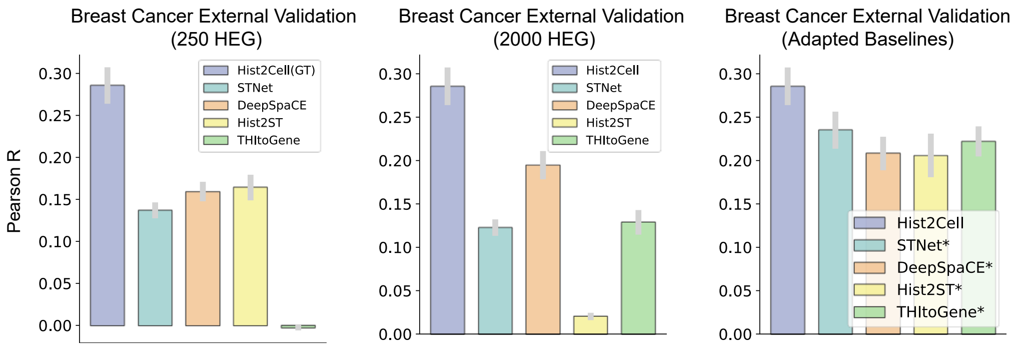

Figure S14. Performance evaluation of cell abundance prediction in the external breast cancer dataset, related to Figure 4

Histogram depicting average Pearson's R values for cell abundance prediction in the external breast cancer dataset. Error bars represent standard error across the cross-validation folds. "250 HEG" and "2000 HEG" represent that, for the previous ST prediction baselines, we follow their setup to first predict the 250/2000 highly expressed genes, and then use the predicted highly expressed genes to estimate the cell abundance. "Adapted Baselines" represents that, for the previous ST prediction baselines, we adapted them to our one-stage prediction strategy to directly predict the fine-grained cell abundances from the histology image. We use * to distinguish the adapted baselines from the original ones. Results show that Hist2Cell demonstrated more accurate predictions of fine-grained cell type abundances across locations, as previous ST prediction baselines suffer from low performance in fine-grained cell type abundances prediction under both "250 HEG" and "2000 HEG" and setups as their noisy and unstable predictions for a relatively small group of genes. When adapted, the performance of previous baselines increases obviously, showing the efficacy of our one-stage prediction strategy.

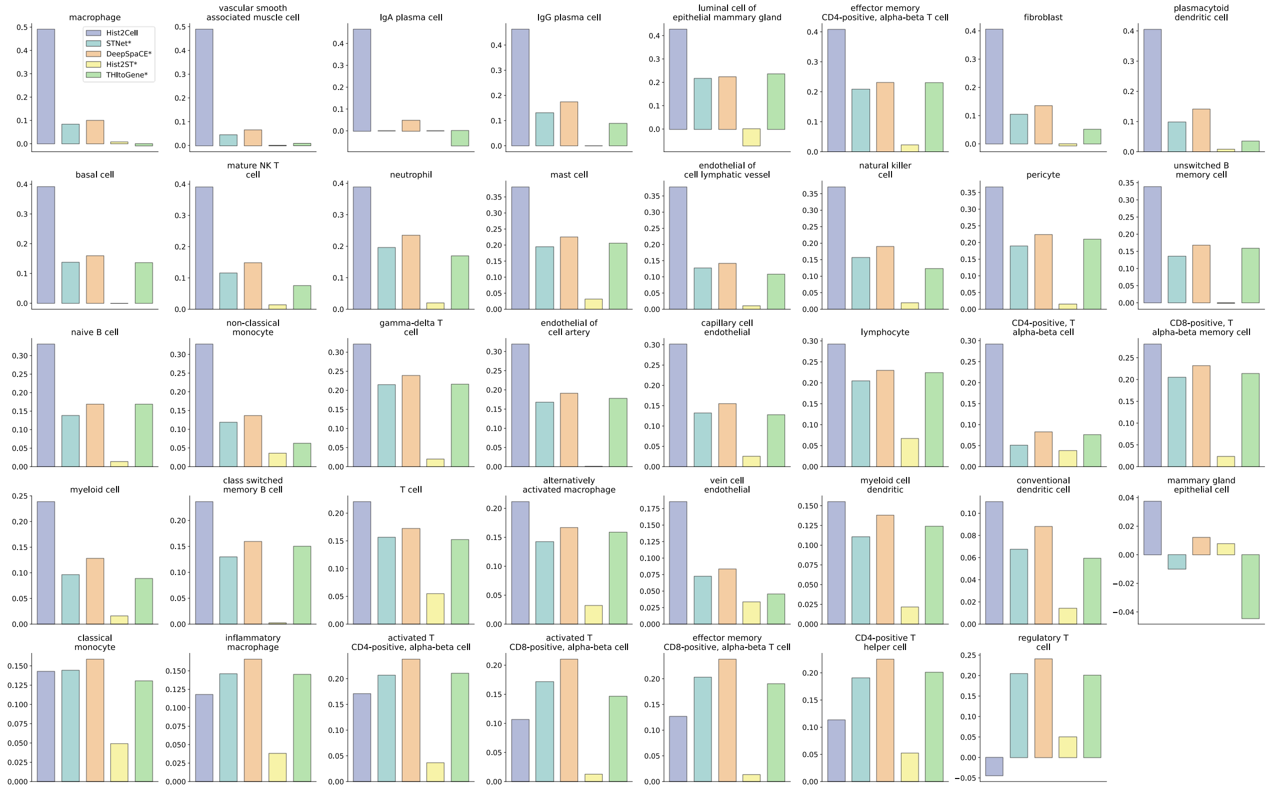

Figure S15. Average Pearson's R comparison of all methods on the external breast cancer validation, related to Figure 4

Histogram representing the average Pearson's R values for all methods of all cell types on the external breast cancer validation.

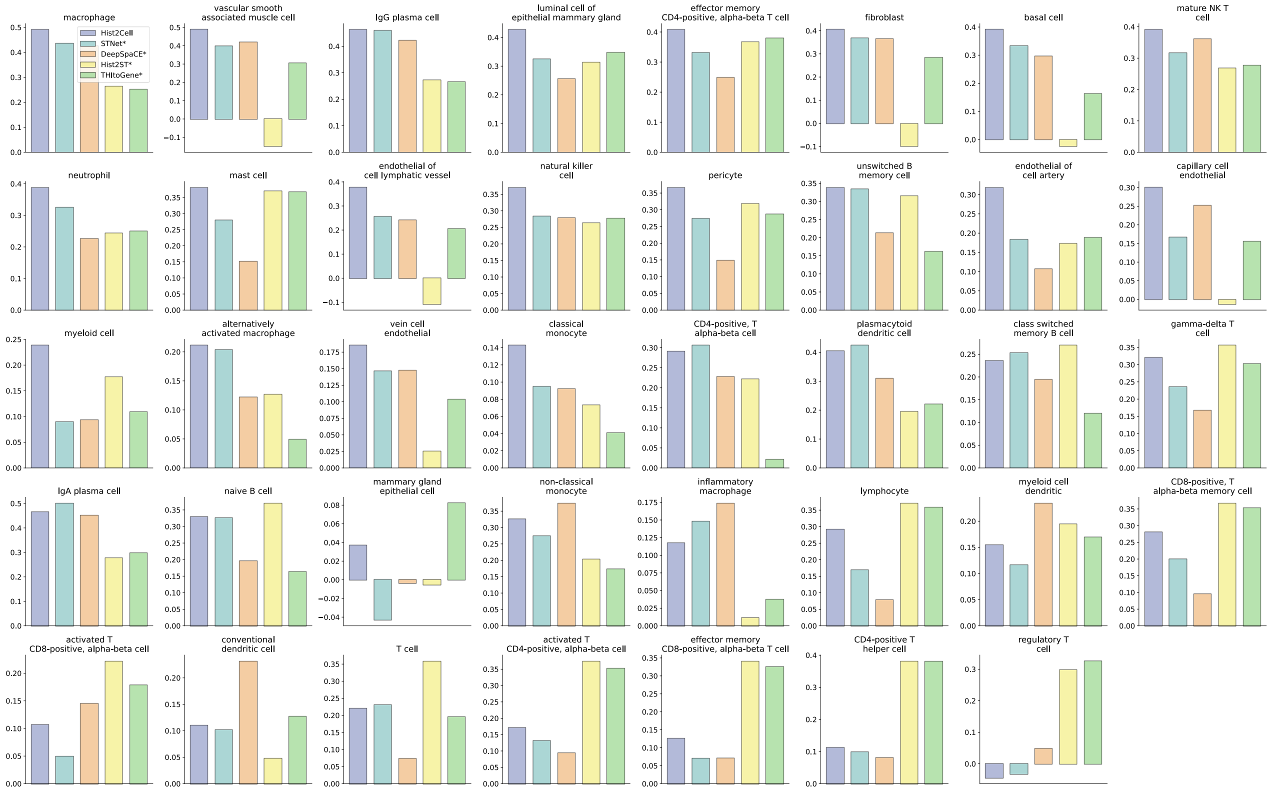

Figure S16. Performance comparison of adapted baseline methods and Hist2Cell on the external breast cancer validation, related to Figure 4

Histogram representing the average Pearson's R values for all adapted (*) baseline methods and Hist2Cell of all cell types on the external breast cancer validation.

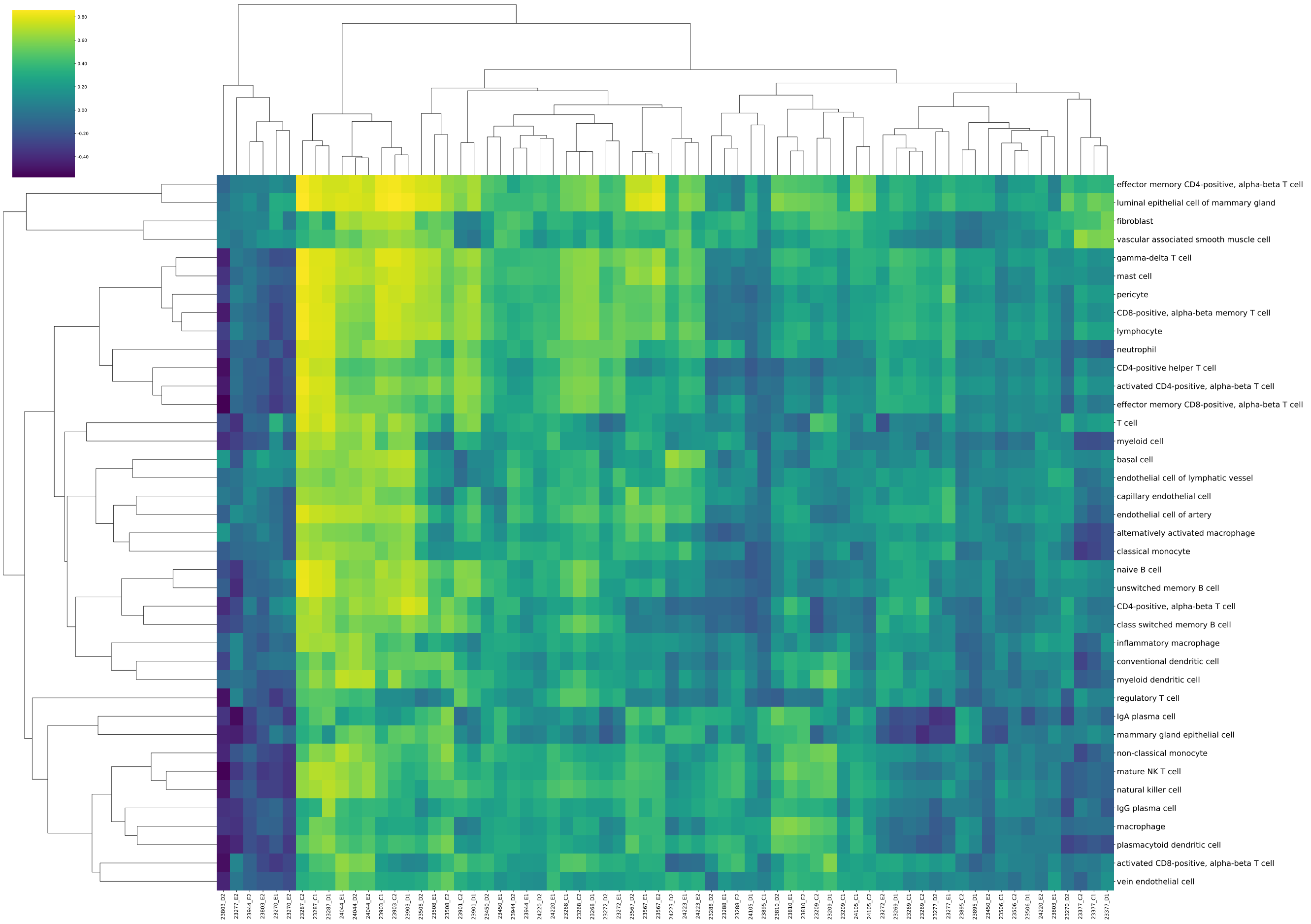

Figure S17. Pearson R Clustermap of Hist2Cell performance on the external breast cancer dataset, related to Figure 4

Clustermap colored by the Pearson R from Hist2Cell for different cell types (y-axis) on different slides (x-axis) during testing in the external breast cancer dataset experiments. Color intensity represents the value of Pearson R. The top 30% cell types across 68 slides have a mean Pearson R 0.54.

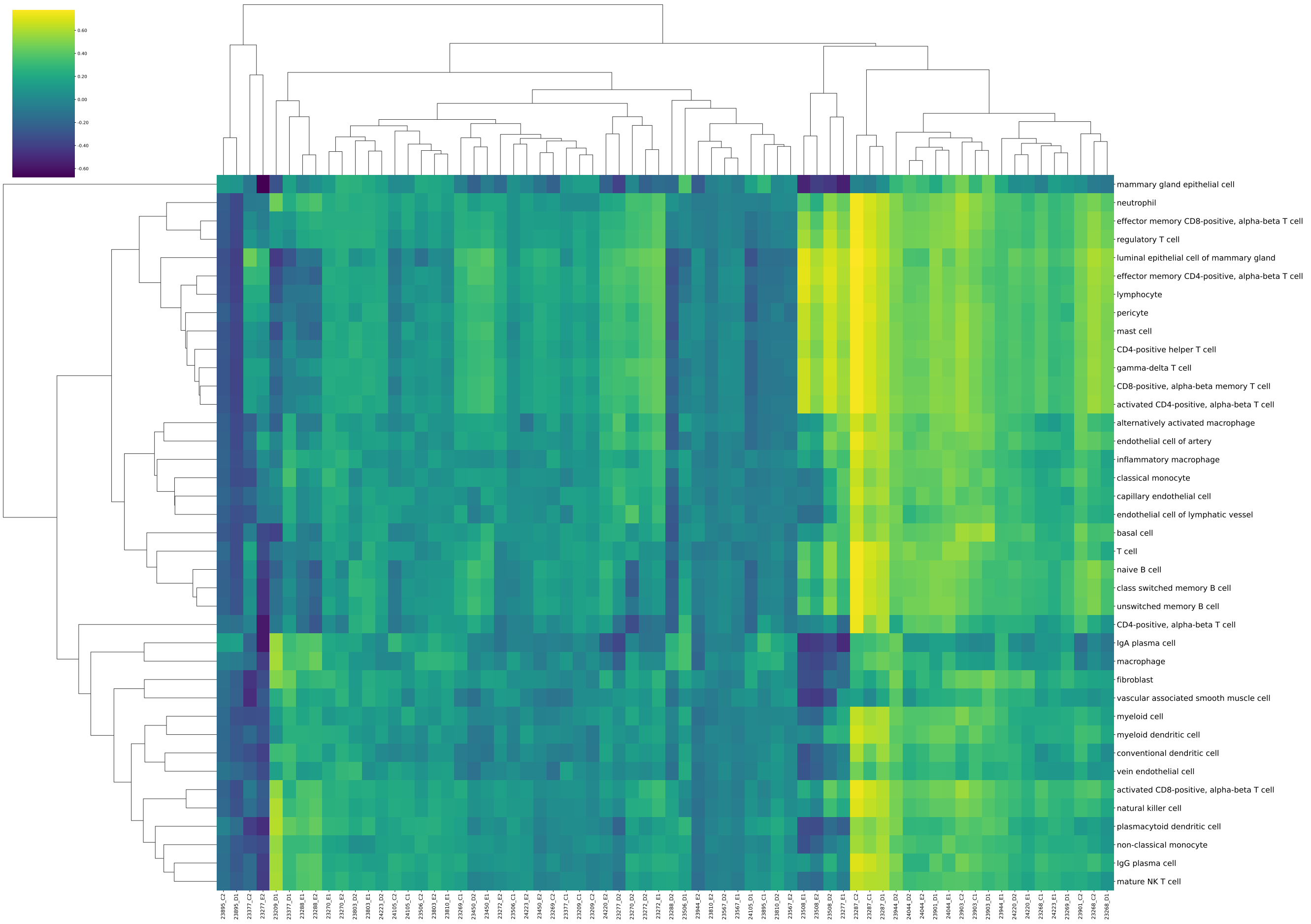

Figure S18. Pearson R Clustermap of DeepSpaCE performance on the external breast cancer dataset, related to Figure 4

Clustermap colored by the Pearson R from DeepSpaCE for different cell types (y-axis) on different slides (x-axis) during testing in the external breast cancer dataset experiments. Color intensity represents the value of Pearson R. The top 30% cell types across 68 slides have a mean Pearson R 0.44.

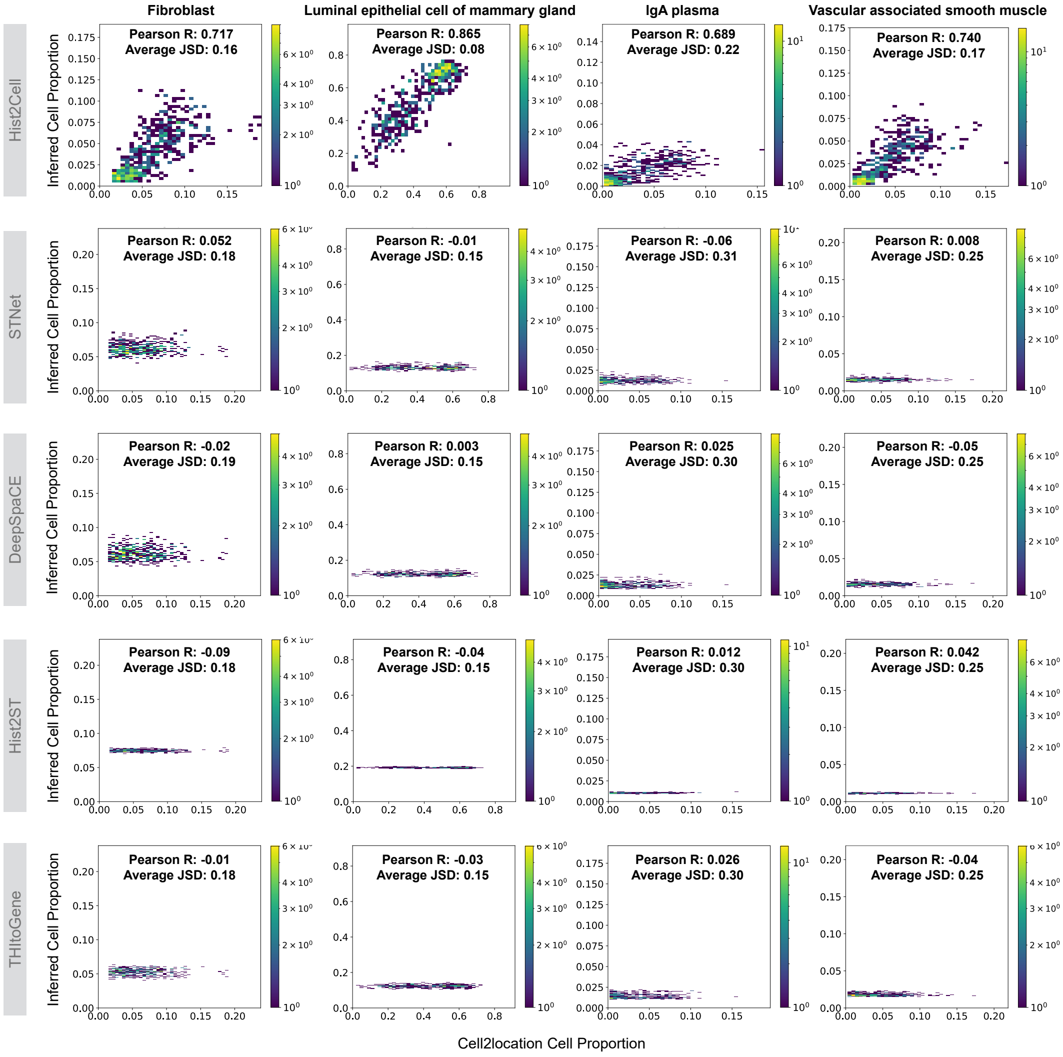

Figure S19. Concordance analysis of Hist2Cell versus baseline methods on external breast cancer tissue, related to Figure 4

2D histogram plots showcasing the concordance of cell abundance between ground truth (x-axis) and prediction of Hist2Cell and the baseline methods (y-axis) across all testing spots in the external breast cancer slide. Color denotes 2D histogram counts. Pearson's R denotes Pearson's correlation coefficient, and JSD denotes Jensen–Shannon divergence. Color intensity represents the spot frequency.

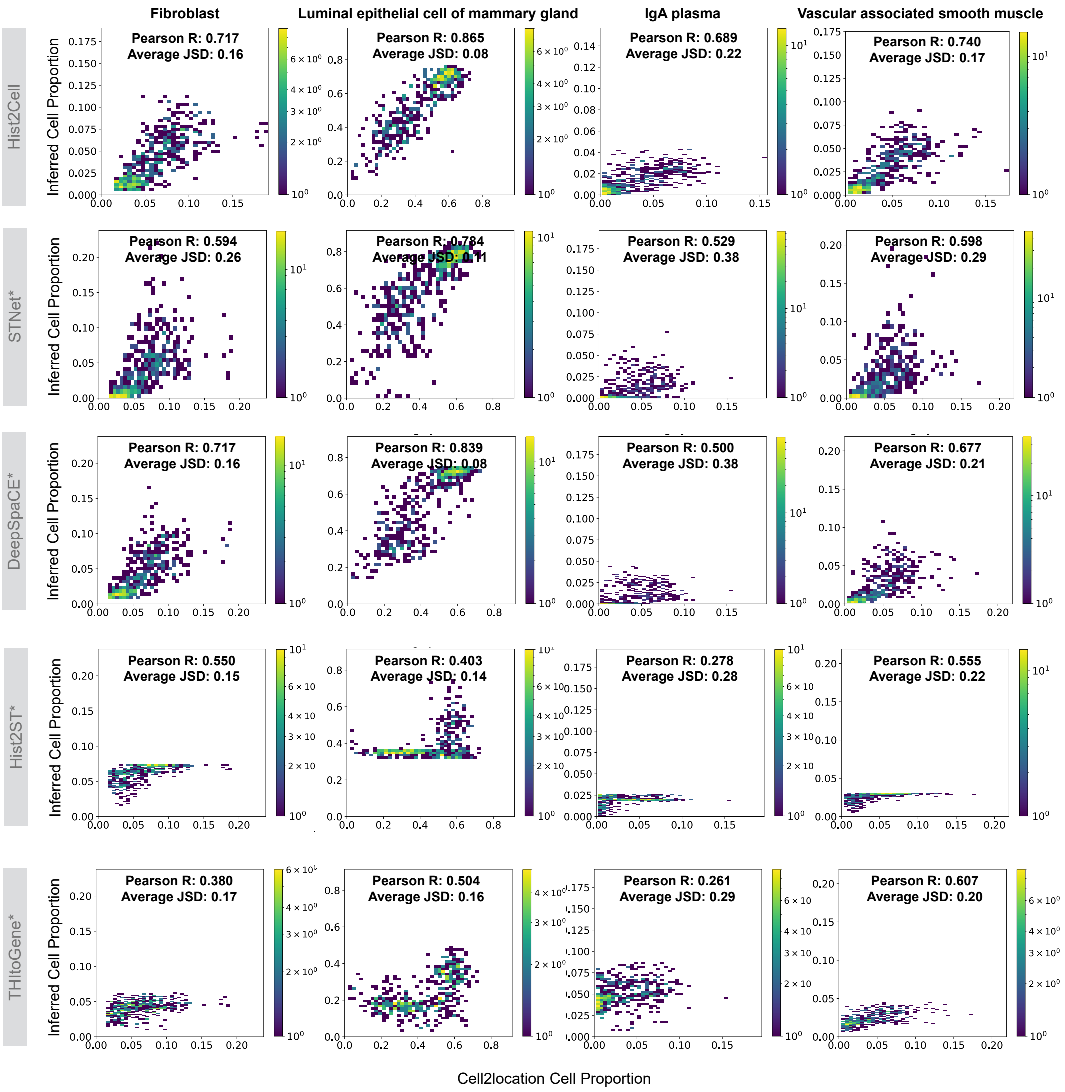

Figure S20. Concordance analysis of Hist2Cell versus adapted baseline methods on external breast cancer tissue, related to Figure 4

2D histogram plots showcasing the concordance of cell abundance between ground truth (x-axis) and prediction of Hist2Cell and the "adapted" (*) baseline methods (y-axis) across all testing spots in the external breast cancer slide. Color denotes 2D histogram counts. Pearson's R denotes Pearson's correlation coefficient, and JSD denotes Jensen–Shannon divergence. Color intensity represents the spot frequency.

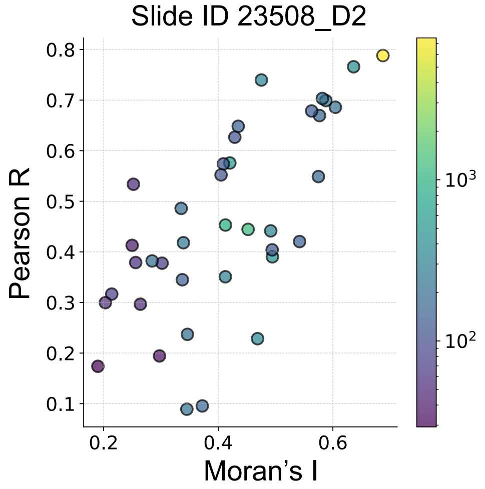

Figure S21. Correlation between spatial auto-correlation and prediction performance, related to Figure 4

Scatter plot for one test slide illustrating the relationship between spatial auto-correlation (Moran's I; x-axis) and Hist2Cell's prediction performance (y-axis) in the healthy human lung dataset. Hist2Cell excelled in predicting cell types exhibiting higher spatial auto-correlation on the external breast cancer dataset. Color intensity represents the spot frequency. The Pearson correlation coefficient between the x-axis and the y-axis is 0.58 in this figure.

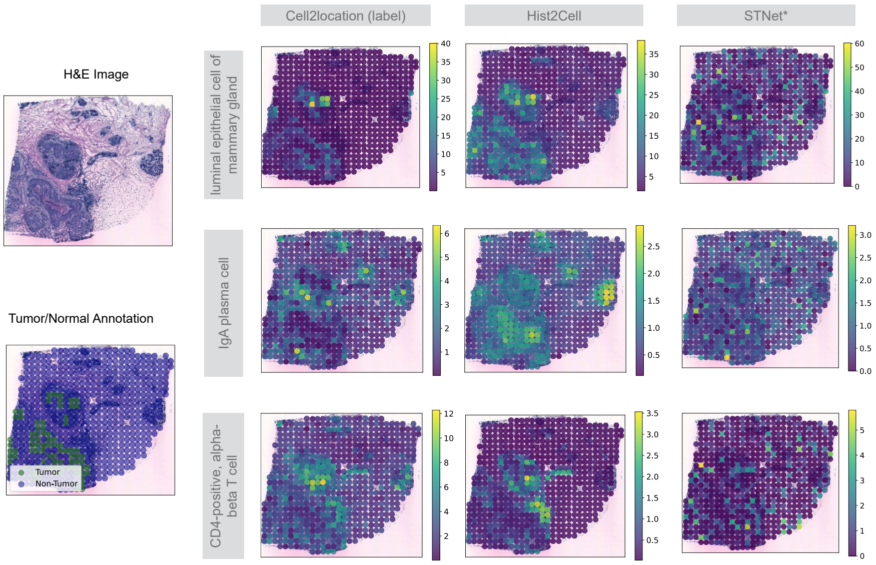

Figure S22. Visual comparison of spatial cell abundance predictions for slide 24044D2, related to Figure 4

The H&E image, expert manual annotation, and the visualizations comparing the spatial key cell abundances as determined by ground truth, Hist2Cell, and STNet* predictions of slide (ID) 24044D2. Hist2Cell shows fewer false positives than STNet*(the best-performing adapted ST prediction baseline). Color intensity represents the cell abundance.

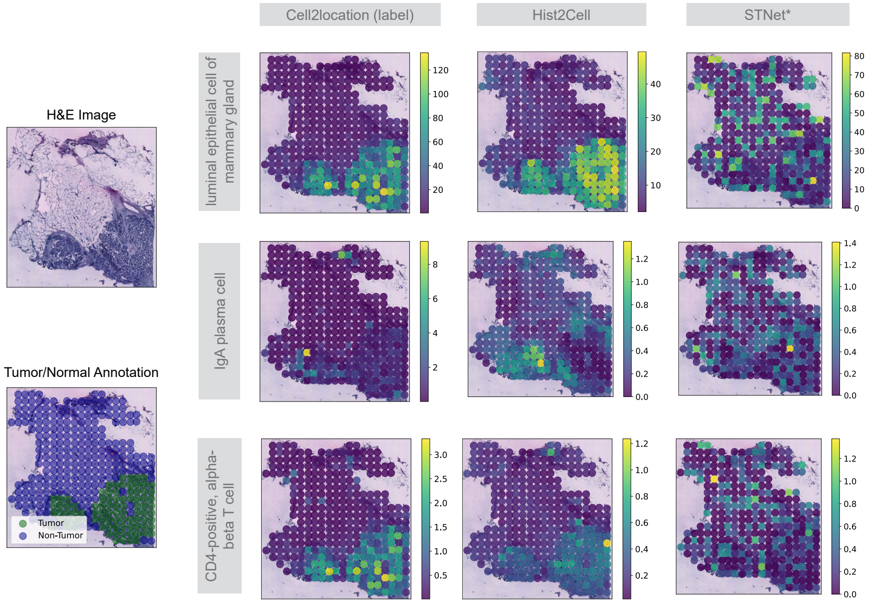

Figure S23. Visual comparison of spatial cell abundance predictions for slide 23287C2, related to Figure 4

The H&E image, expert manual annotation, and the visualizations comparing the key spatial cell abundances as determined by ground truth, Hist2Cell, and STNet* predictions of slide (ID) 23287C2. Hist2Cell shows fewer false positives than STNet*(the best-performing adapted ST prediction baseline). Color intensity represents the cell abundance.

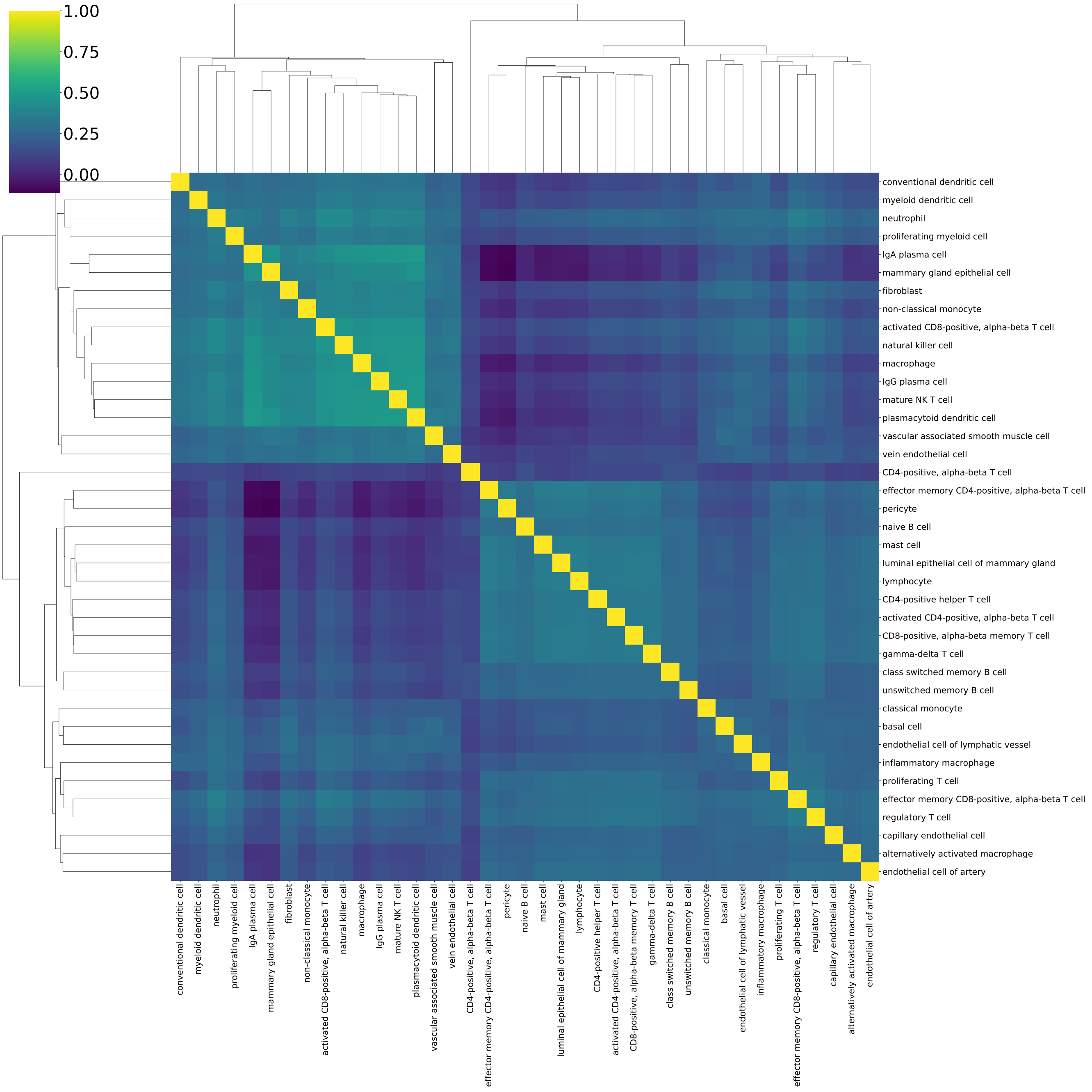

Figure S24. Detailed Moran's R Clustermaps of ground truth cell abundances in human breast cancer tissue, related to Figure 4

Detailed Human breast cancer Moran's R Clustermaps calculated from ground truth cell type abundances (cell2location algorithm) in Figure 4F. Color intensity represents the value of Moran's R.

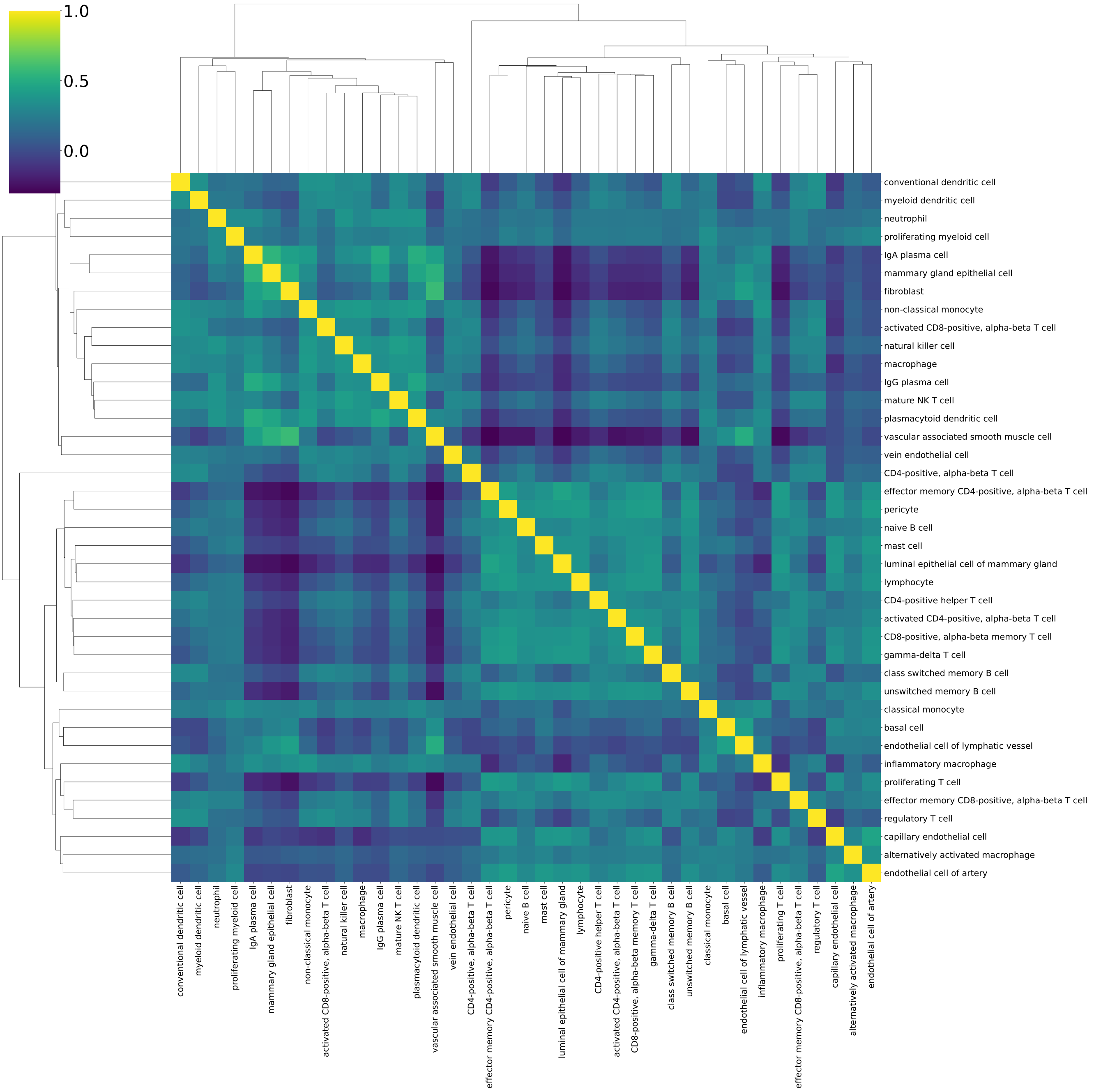

Figure S25. Detailed Moran's R Clustermaps of Hist2Cell predictions in human breast cancer tissue, related to Figure 4

Detailed Human breast cancer Moran's R Clustermaps calculated from Hist2Cell predictions in Figure 4F. Color intensity represents the value of Moran's R.

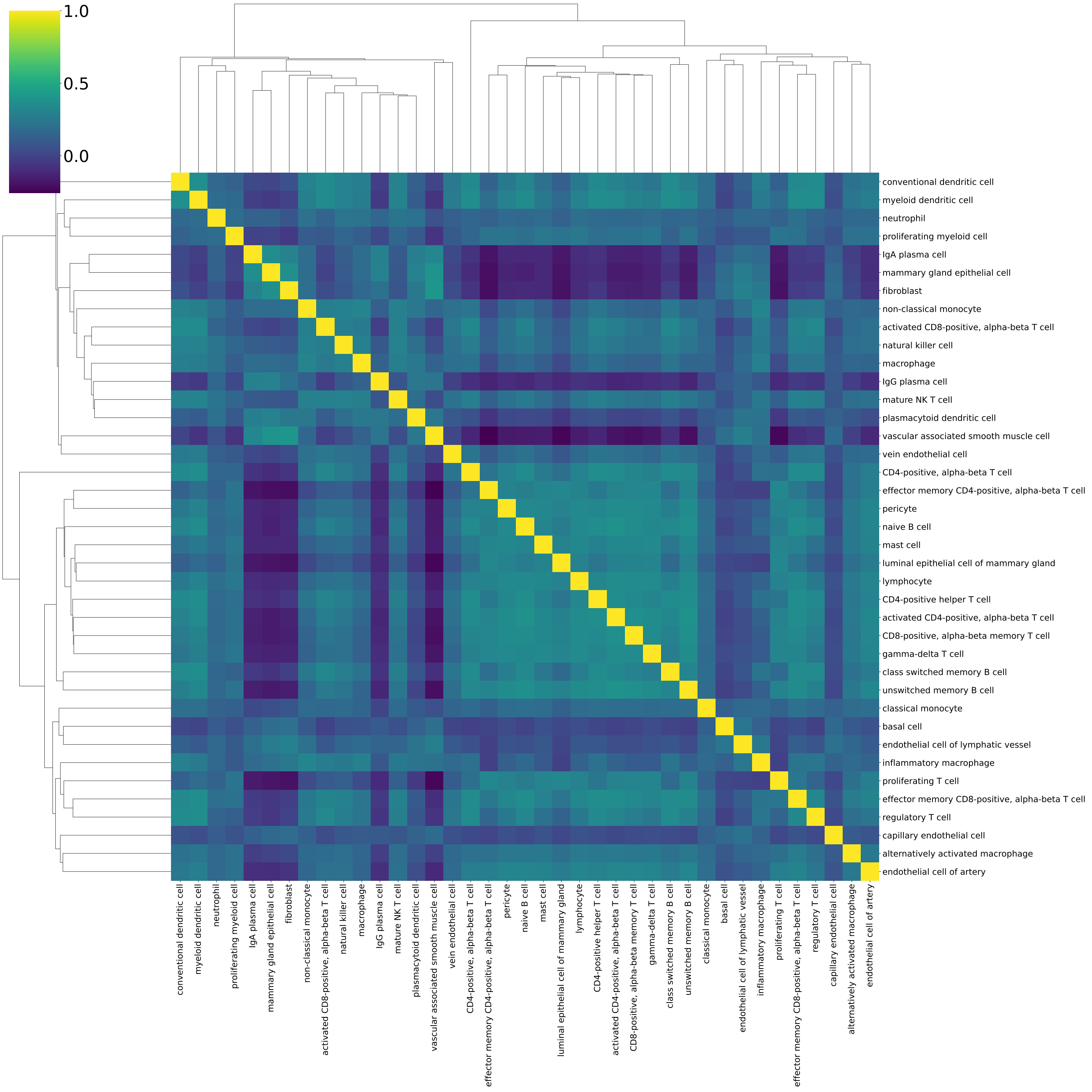

Figure S26. Detailed Moran's R Clustermaps of STNet* predictions in human breast cancer tissue, related to Figure 4

Detailed Human breast cancer Moran's R Clustermaps calculated from STNet* predictions in Figure 4F. Color intensity represents the value of Moran's R.

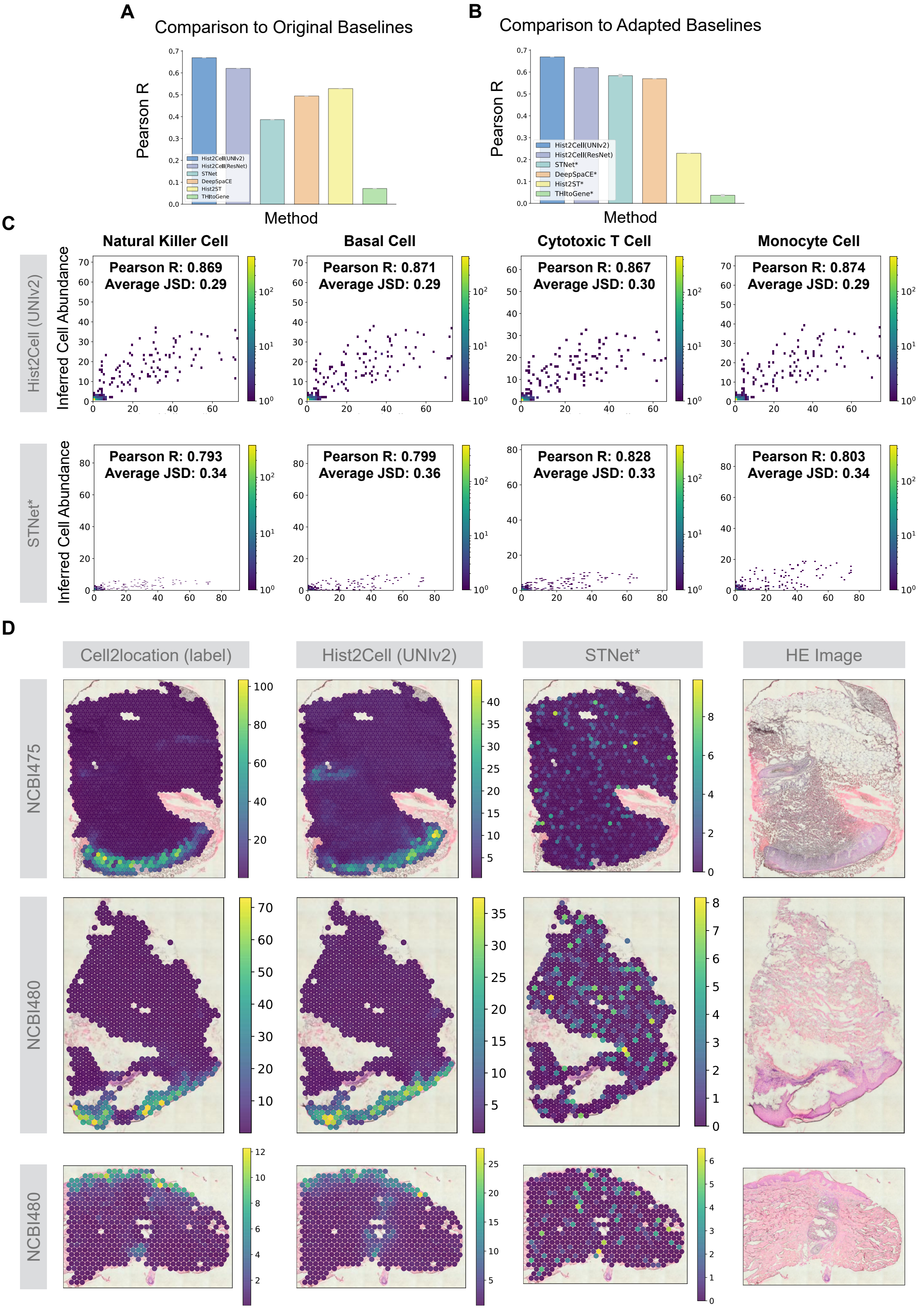

Figure S27. Performance evaluation of Hist2Cell on the skin disease dataset, related to Figure 4

(A, B) Histogram depicting the average Pearson's R values for cell abundance prediction for the skin disease dataset. An asterisk * denotes that we adapted the corresponding ST-prediction-based baseline to our one-stage strategy to predict the fine-grained cell abundances. Error bars

represent standard error across the cross-validation folds.
(C) 2D histogram plots showcasing the concordance of cell abundance between ground truth (x-axis) and Hist2Cell's prediction (y-axis) across all testing spots in the skin disease slide. Color denotes 2D histogram counts, and JSD denotes Jensen–Shannon divergence. Color intensity represents the spot frequency.
(D) The H&E images and the visualisations comparing the natural killer cell abundances as determined by ground truth, Hist2Cell, and STNet* predictions. Color intensity represents the cell abundance.

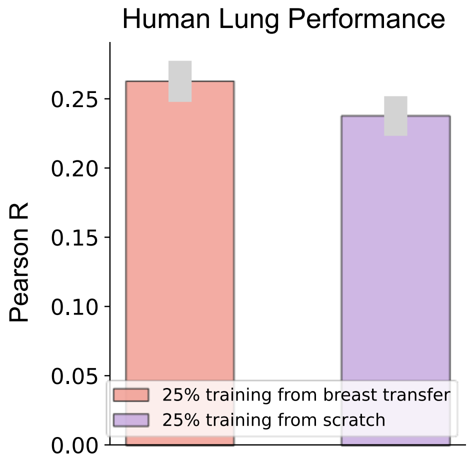

Figure S28. Transfer learning performance on the human lung dataset under low data regimes, related to Figure 4

Histogram depicting the average Pearson's R values for cell abundance prediction for the human lung dataset under low data regime (25% training data). Error bars represent standard error across the cross-validation folds. Training from breast transfer denotes that we use the model weights trained on the breast cancer dataset as the initialization. Results show that the knowledge of predicting fine-grained cell type abundances could be transferred across different tissues via the pre-trained model parameters.

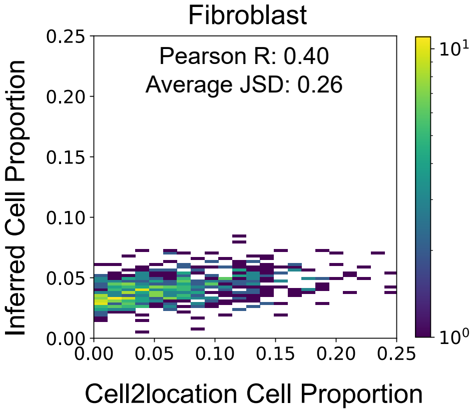

Figure S29. Concordance analysis of fibroblast cell proportion predictions, related to Figure 5

2D histogram plots showing the concordance between the ground truth cell proportion (x-axis) and the cell proportion predicted by Hist2Cell (y-axis) for fibroblast cells across all cases in the TCGA breast cancer cohort. Color intensity represents the spot frequency.

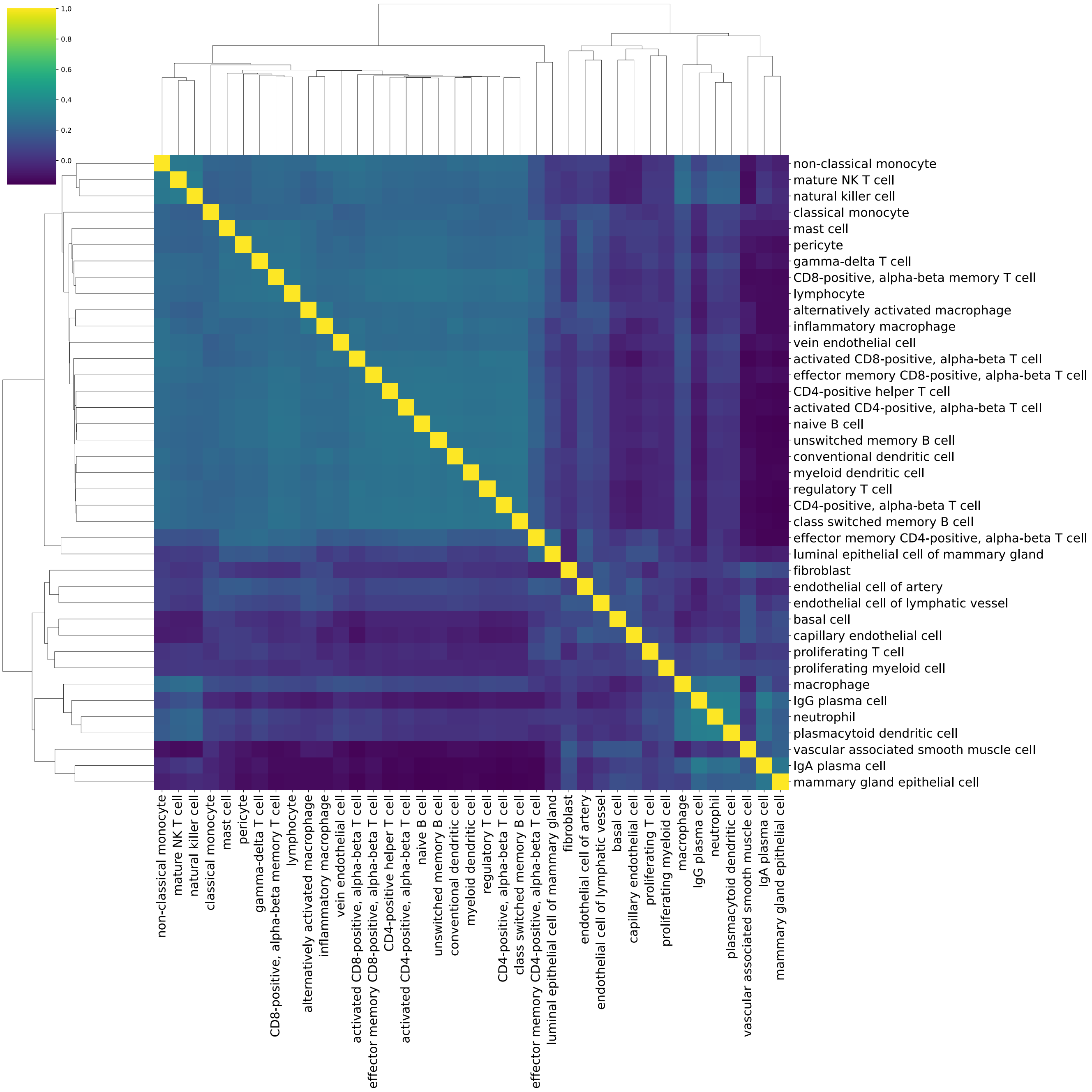

Figure S30. Detailed Moran's R Clustermaps of Hist2Cell predictions in the TCGA breast cancer cohort, related to Figure 5

Detailed Human breast cancer Moran's R Clustermaps calculated from Hist2Cell predictions in Figure 5E. Color intensity represents the value of Moran's R.

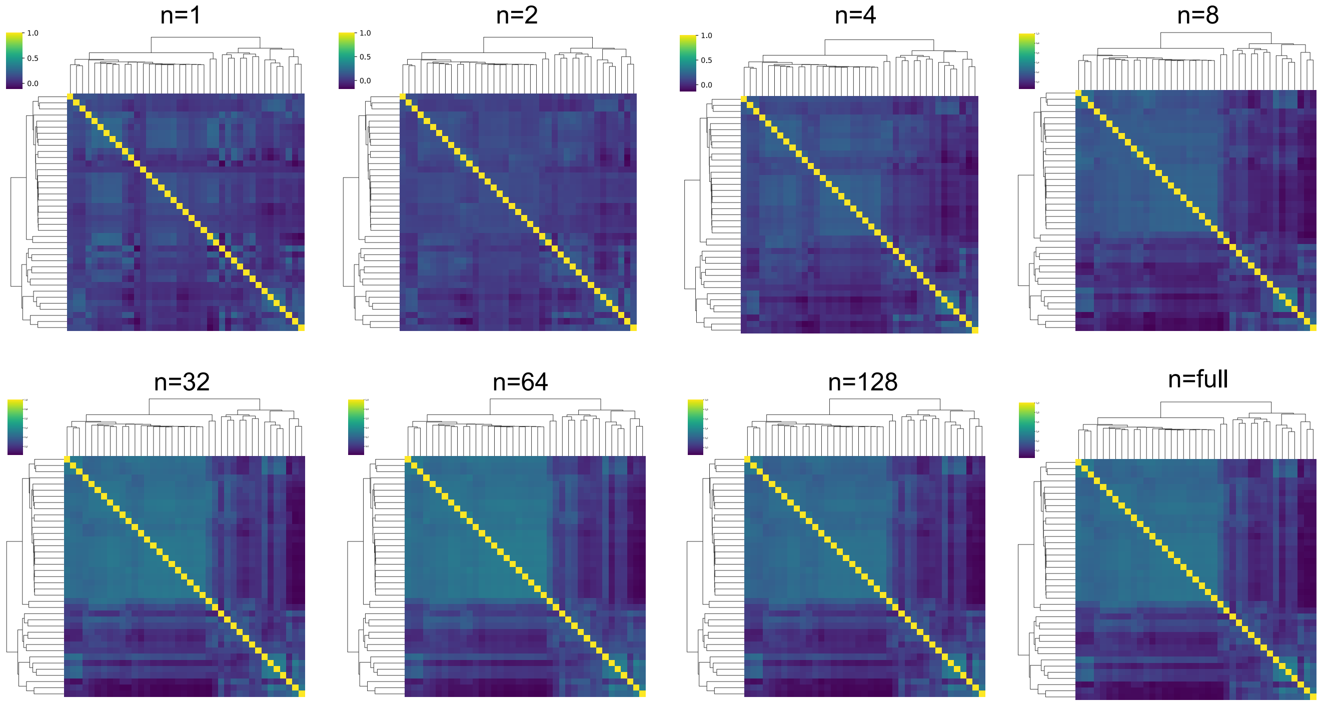

Figure S31. Hist2Cell provides more stabilized consensus analysis with larger sample sizes, related to Figure 5

Consensus clustermaps generated by Hist2Cell using varying numbers of samples from the TCGA breast cancer cohort. n denotes the number of randomly sampled slides, with $n =$ full indicating the use of the entire cohort. The consensus clustermaps become more stable as the number of sampled slides increases. Color intensity represents the value of Moran's R.

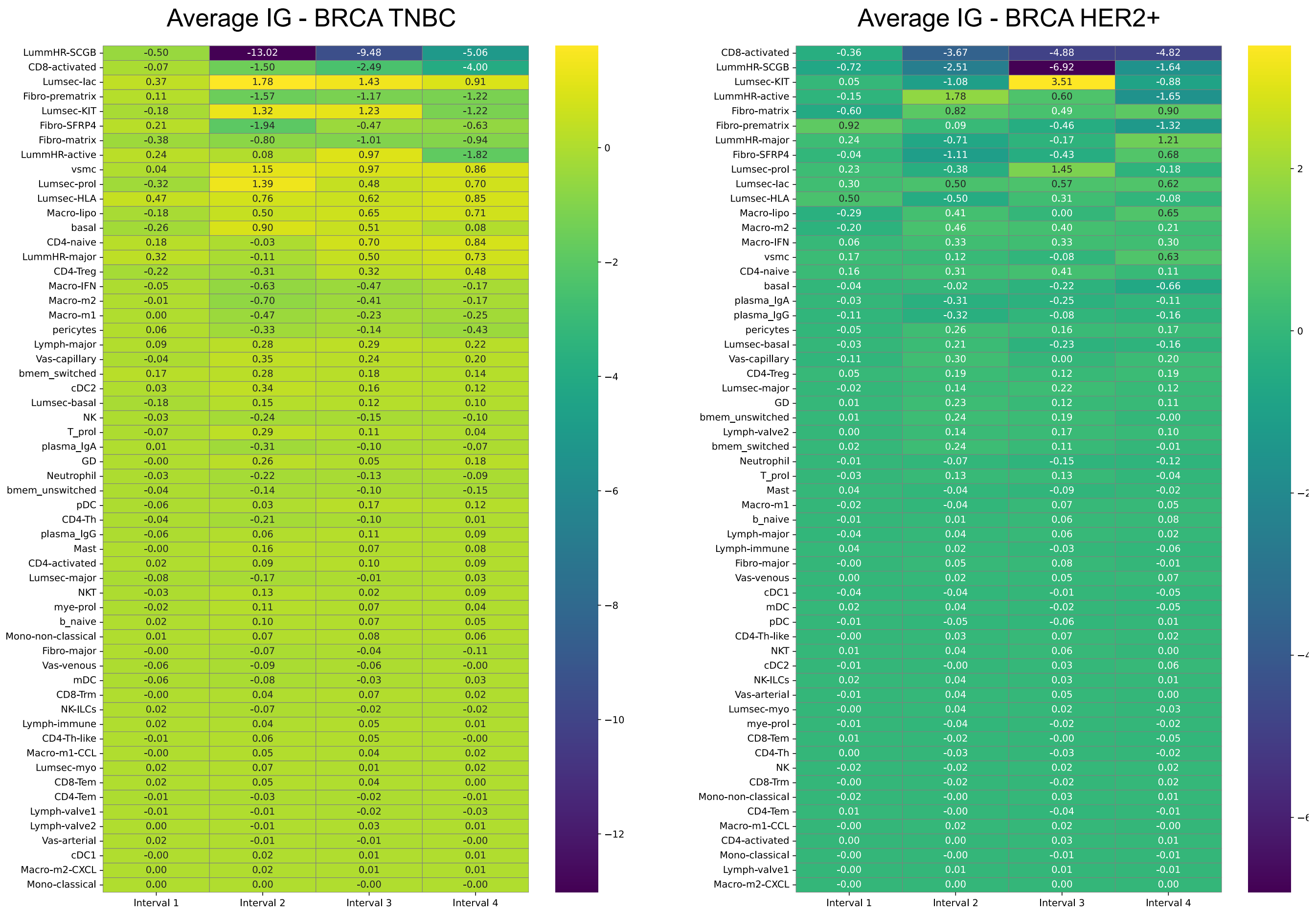

Figure S32. Average integrated gradients for survival analysis of BRCA TNBC and BRCA HER2+, related to Figure 5

Average integrated gradients (representing the feature attribution of the Hist2Cell-prediction-based Cox regression model) for the survival analysis of BRCA TNBC and BRCA HER2+. The Y-axis is the different fine-grained transcriptional cell types and the X-axis is the four survival intervals ranging from shortest to longest survival time among the patients. The fine-grained transcriptional cell types are ranked according to their importance (mean value) among four intervals. Color intensity represents the value of the average integrated gradients. Interesting observations could be found: (1) in the ten 10 major cell types identified by the original study of the scRNA-seq reference[46], luminal hormone-responsive (LumHR-), luminal secretory (LumSec-), fibroblasts (fibro-) and immune (CD8-activated) cells play important roles (on the top of the ranked heatmap) in predicting patients' mortality while other cell types, like the vascular (vas-venous) and the rare cells, less related (on the lower part of the ranked heatmap) to the patient's survival status, this aligns with the existing studies analyzing breast cancer survival[47–52]; (2) the effect of a certain cell type might vary between short-time and long-time survival analysis, for instance, we note that CD8-activated cells will have a stronger effect for long-time survival (4 times bigger average integrated gradients for later survival intervals) for HER2+ cancers, such observation provides potential biological insights for future cancer research and probably motivates customized BRCA-HER2+ treatment plan to promote the proliferation of certain cell types.

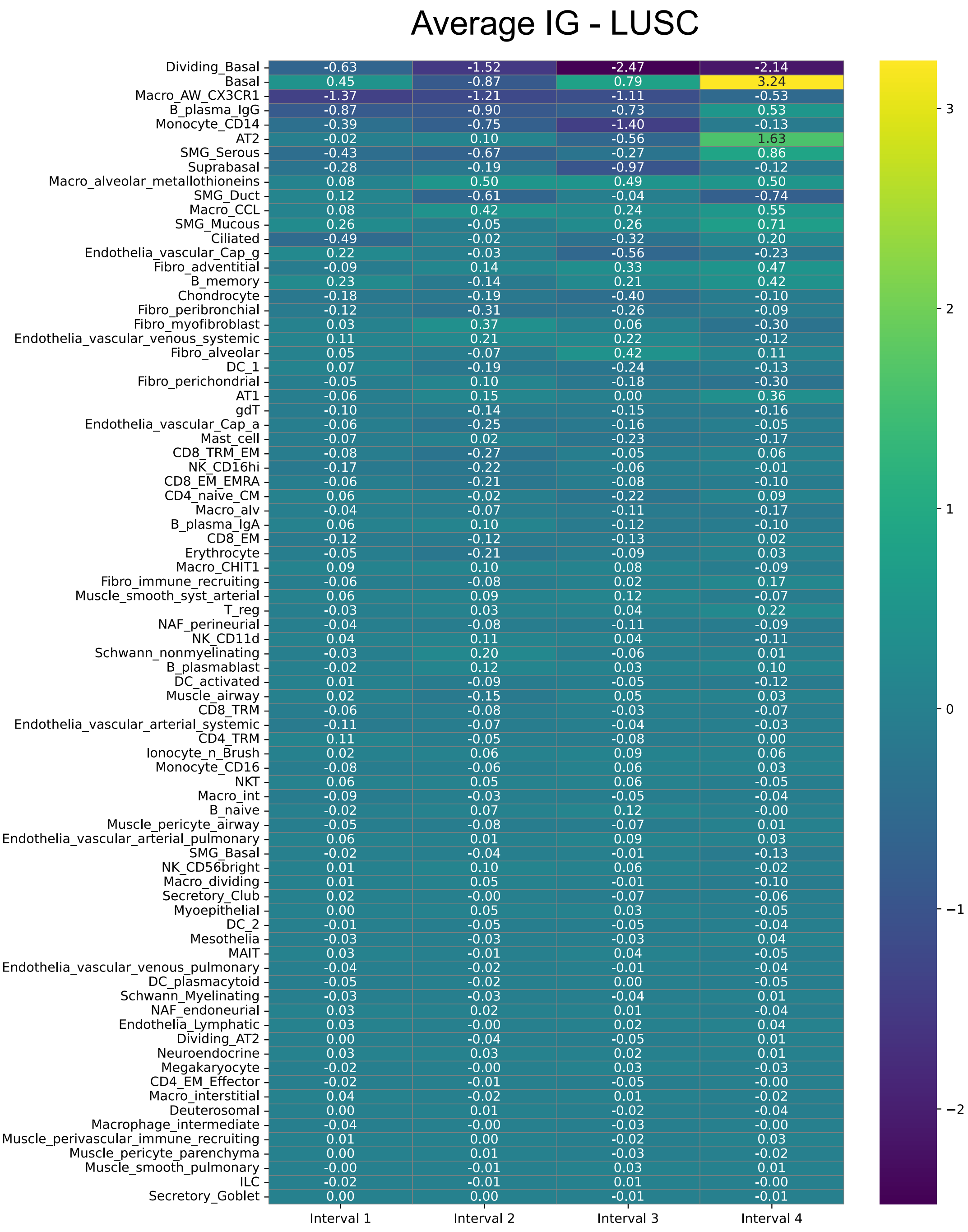

Figure S33. Average integrated gradients for survival analysis of LUSC, related to Figure 5

Average integrated gradients (representing the feature attribution of the modelname-prediction-based Cox regression model) for the survival analysis of LUSC. The Y-axis is the different fine-grained transcriptional cell types and the X-axis is the four survival intervals ranging from shortest to longest survival time among the patients. The fine-grained transcriptional cell types are ranked according to their importance (mean value) among four intervals. Color intensity represents the value of the average integrated gradients.

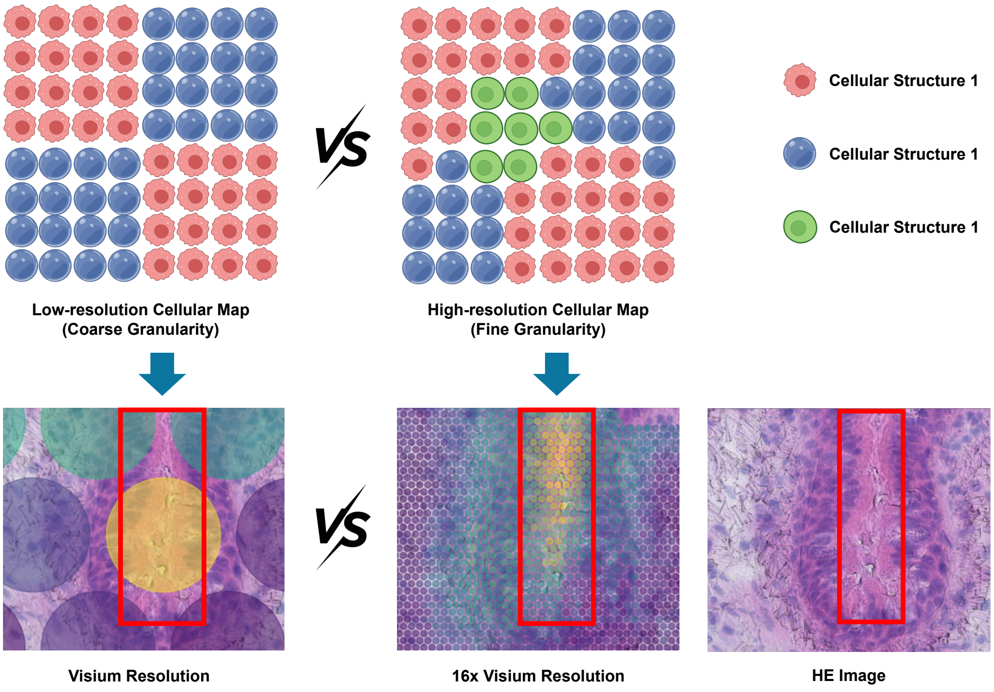

Figure S34. Super-resolution reveals fine-scale cellular architecture missed by low-resolution methods, related to Figure 6

This figure shows the predicted ciliated cell abundance from our human lung super-resolution experiment. Low-resolution predictions (bottom left) are averaged signals that cannot distinguish between different micro-architectures or locate individual, sparsely dispersed cells. In contrast, our super-resolution approach (bottom middle) reconstructs these finer structural details by predicting at multiple sub-locations, resolving this ambiguity and providing a more faithful view of the tissue's organization. Color intensity represents the cell abundance.

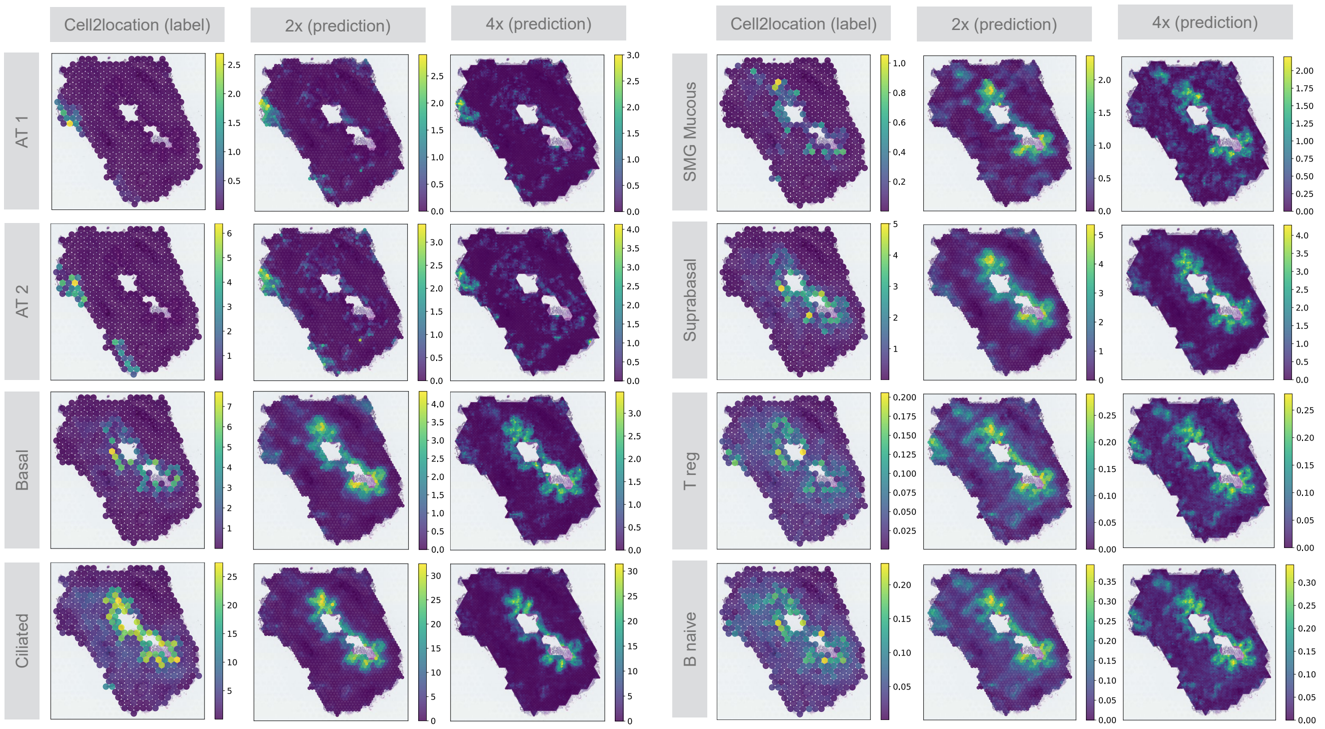

Figure S35. Visualization of super-resolution capabilities at varying scales, related to Figure 6

Visualizations comparing low-resolution ground truth cell abundance with directly predicted $2\times$ and $4\times$ high-resolution results by Hist2Cell. Higher resolutions provide more detailed mapping in concordance with the manual annotation shown in Figure 3B. Color intensity represents the cell abundance.

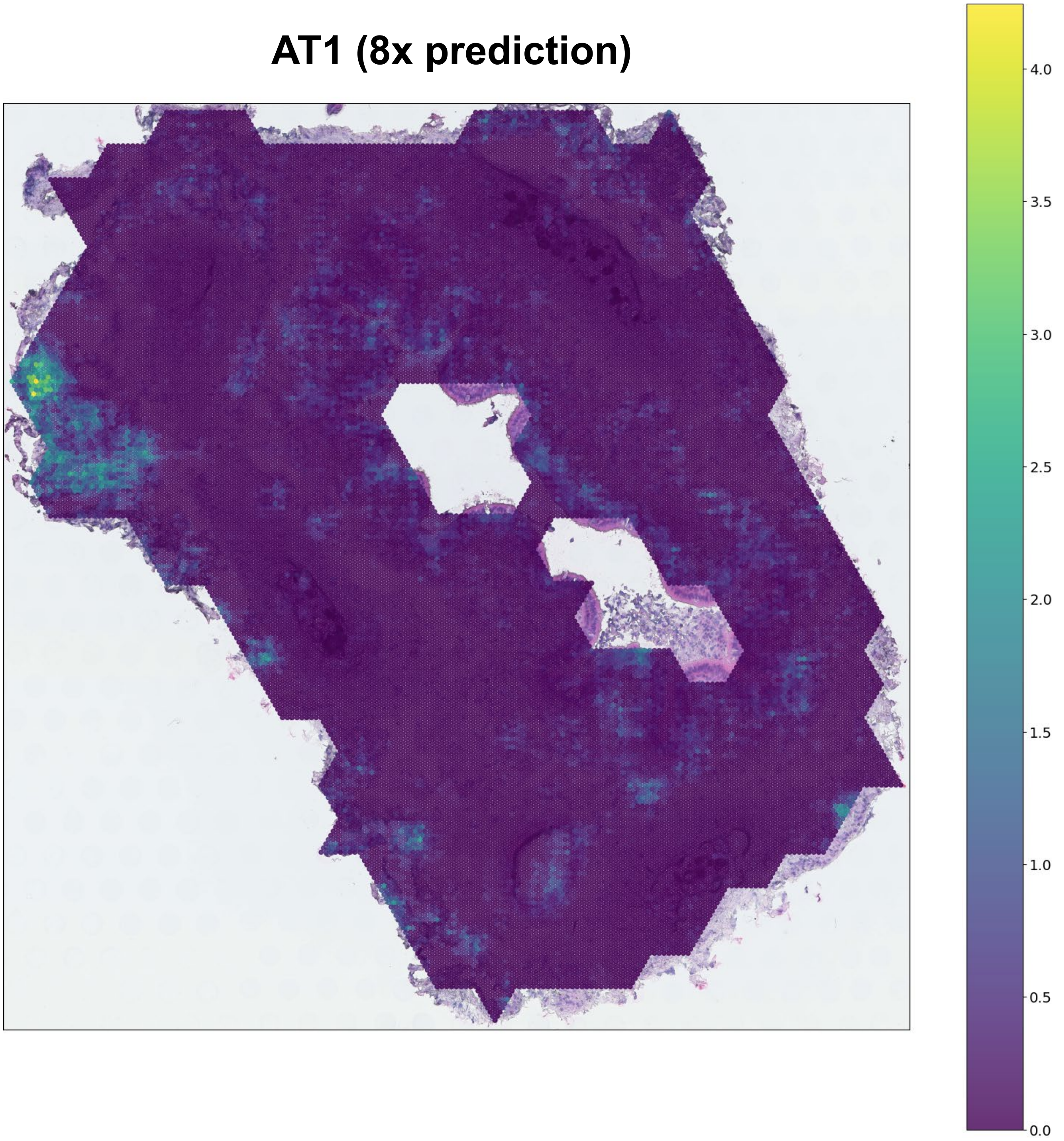

Figure S36. Visualization of 8× high-resolution prediction for AT1 cells, related to Figure 6

Visualization of the directly predicted 8× high-resolution results of AT1 cells by Hist2Cell. Color intensity represents the cell abundance.

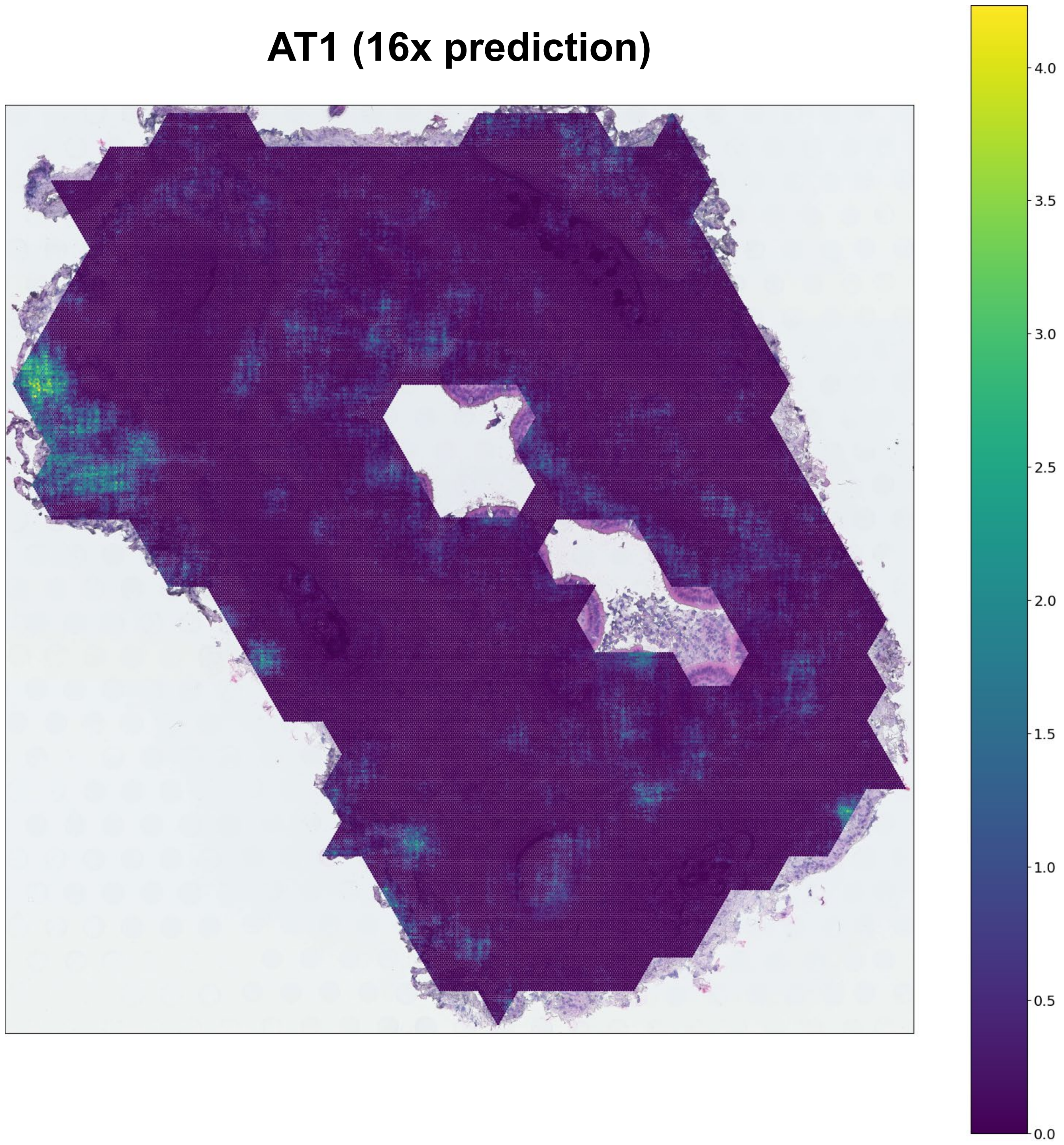

Figure S37. Visualization of 16× high-resolution prediction for AT1 cells, related to Figure 6

Visualization of the directly predicted 16× high-resolution results of AT1 cells by Hist2Cell. Color intensity represents the cell abundance.

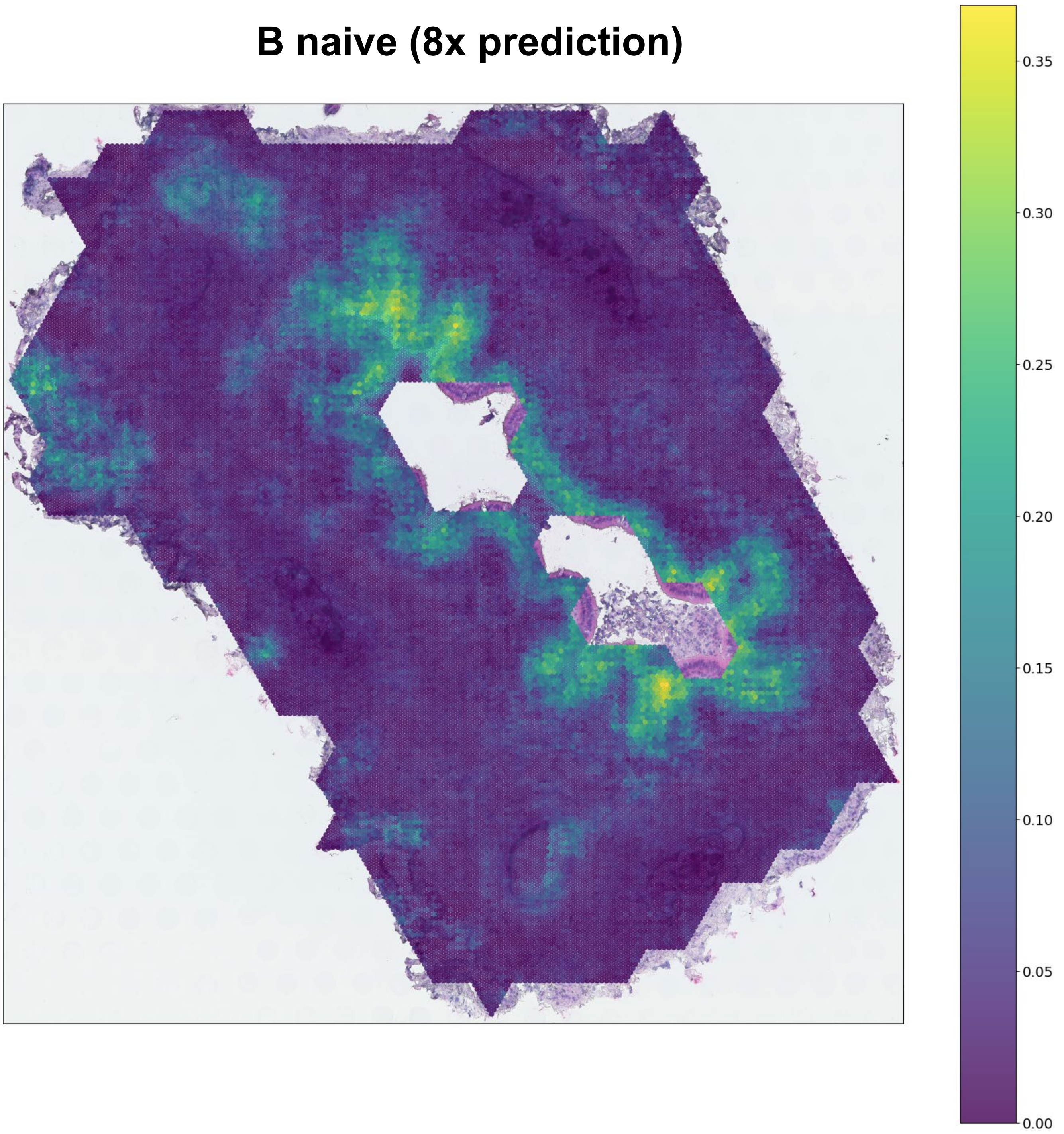

Figure S38. Visualization of 8× high-resolution prediction for B naive cells, related to Figure 6

Visualization of the directly predicted 8× high-resolution results of B naive cells by Hist2Cell. Color intensity represents the cell abundance.

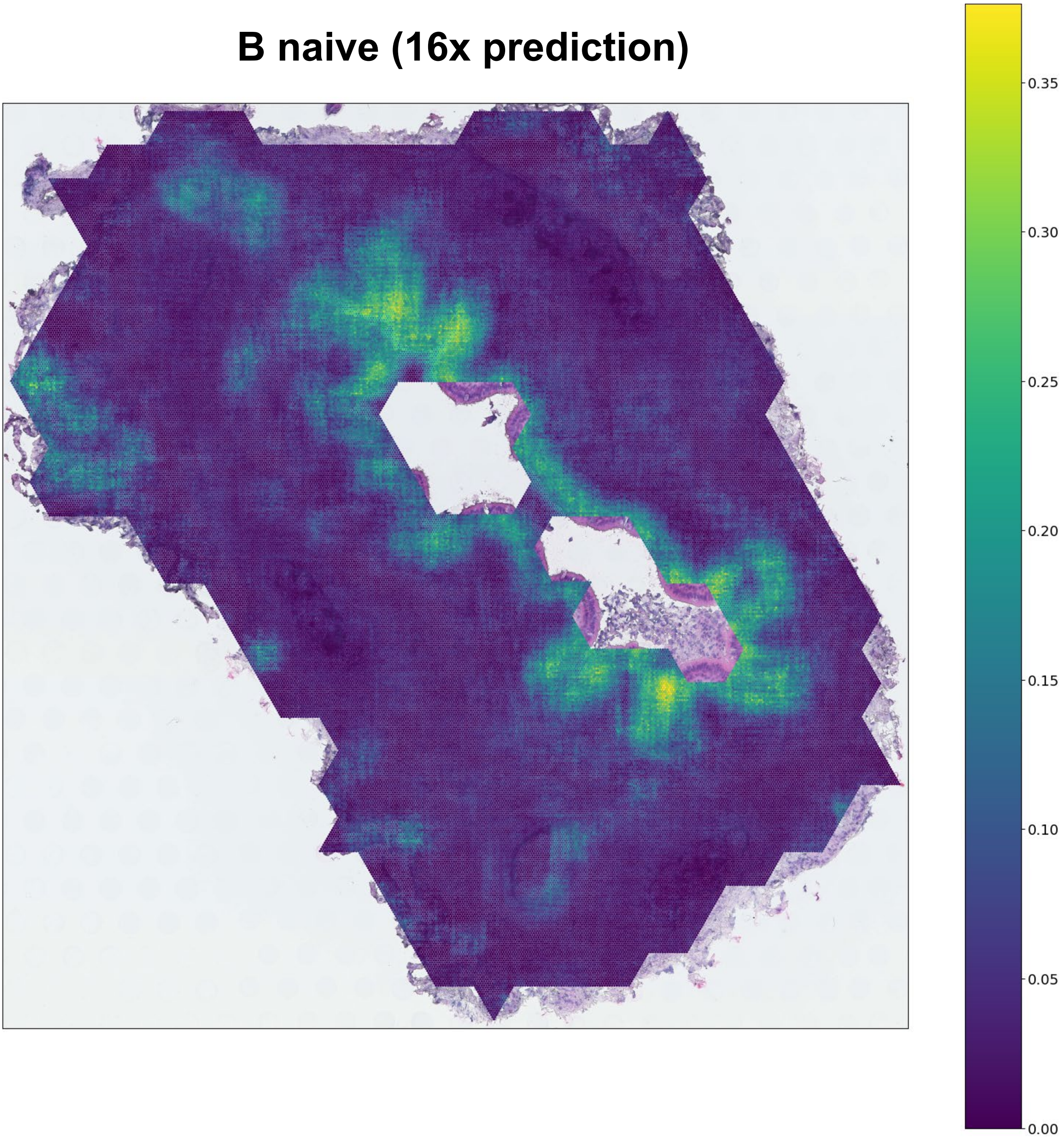

Figure S39. Visualization of 16× high-resolution prediction for B naive cells, related to Figure 6
Visualization of the directly predicted 16× high-resolution results of B naive cells by Hist2Cell. Color intensity represents the cell abundance.

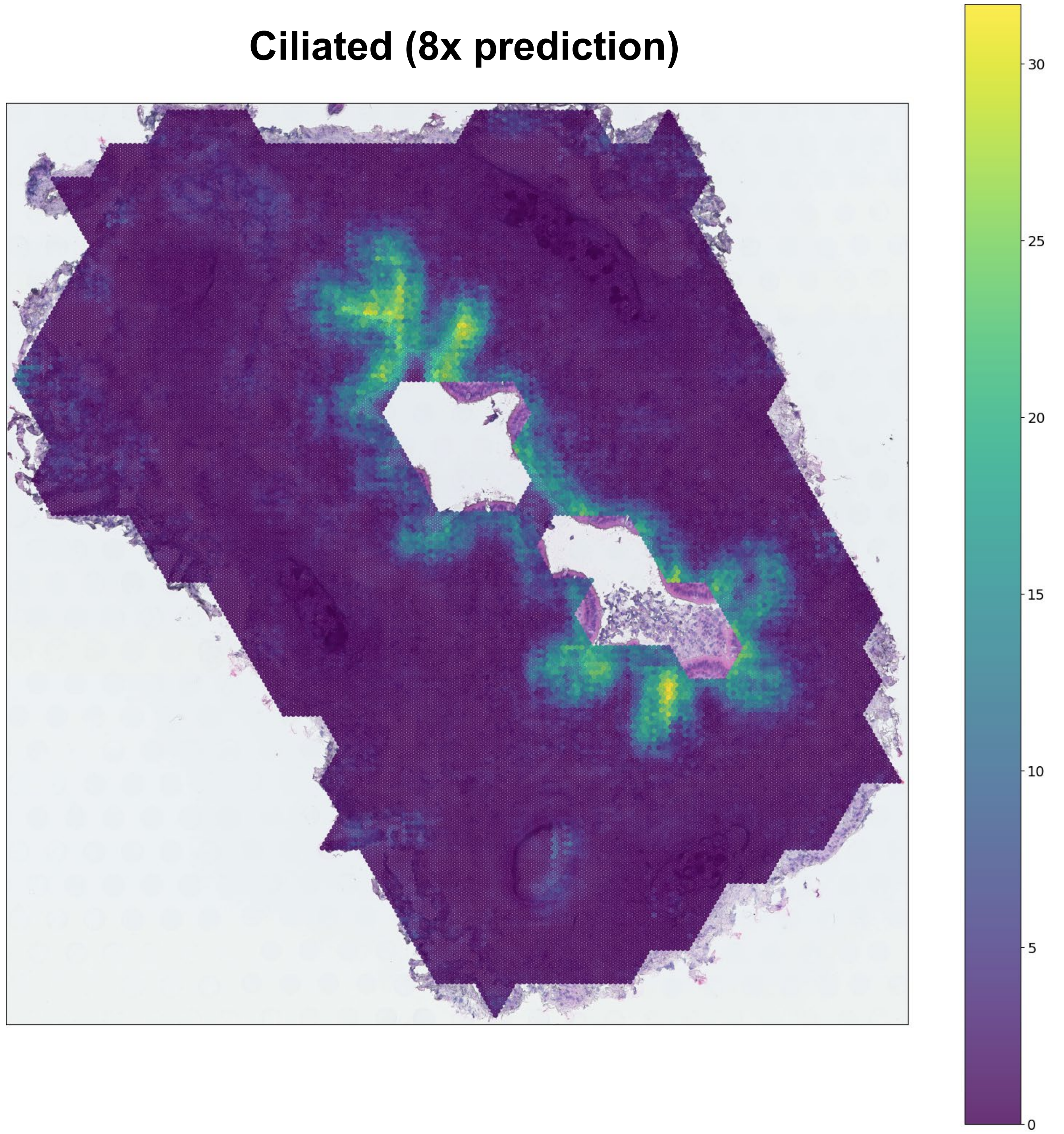

Figure S40. Visualization of 8× high-resolution prediction for Ciliated cells, related to Figure 6

Visualization of the directly predicted 8× high-resolution results of Ciliated cells by Hist2Cell. Color intensity represents the cell abundance.

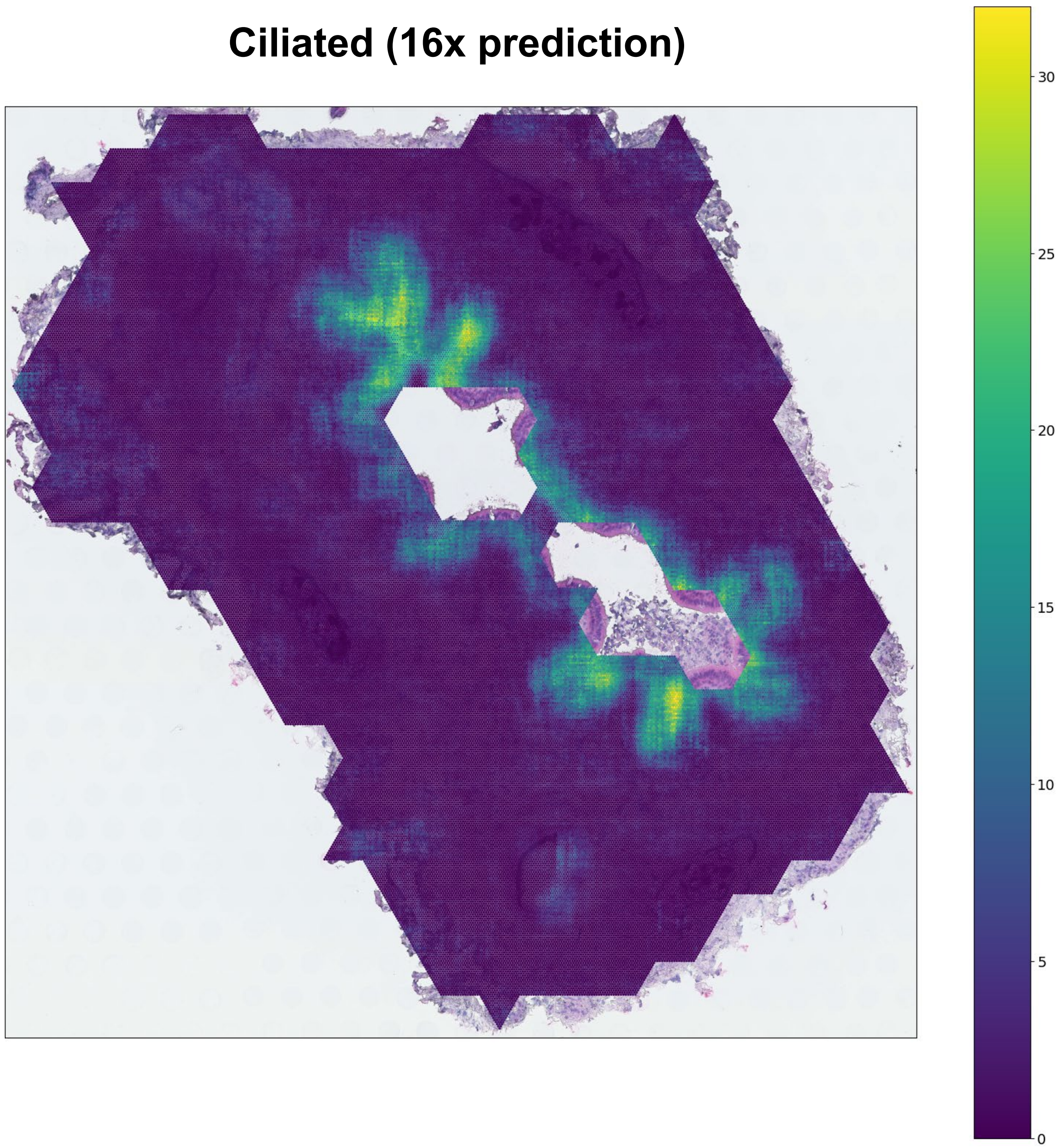

Figure S41. Visualization of 16× high-resolution prediction for Ciliated cells, related to Figure 6

Visualization of the directly predicted 16× high-resolution results of Ciliated cells by Hist2Cell. Color intensity represents the cell abundance.

Ciliated (16x prediction zoom in)

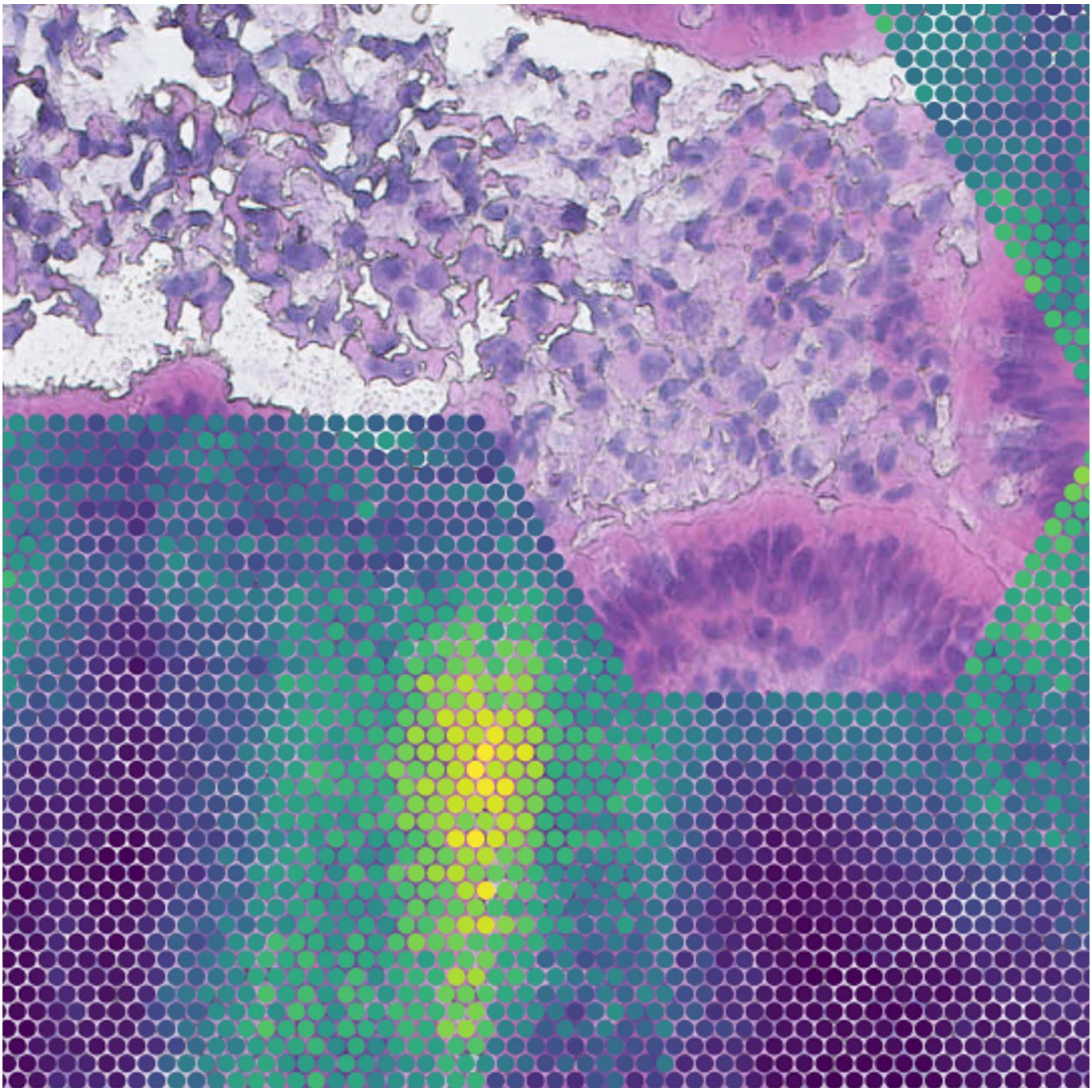

Figure S42. Zoom-in visualization of 16× super-resolved Ciliated cells, related to Figure 6

The zoom-in visualization of the directly predicted 16× high-resolution results of Ciliated cells by Hist2Cell, the sub-spot size is close and even smaller than a single cell. Color intensity represents the cell abundance.

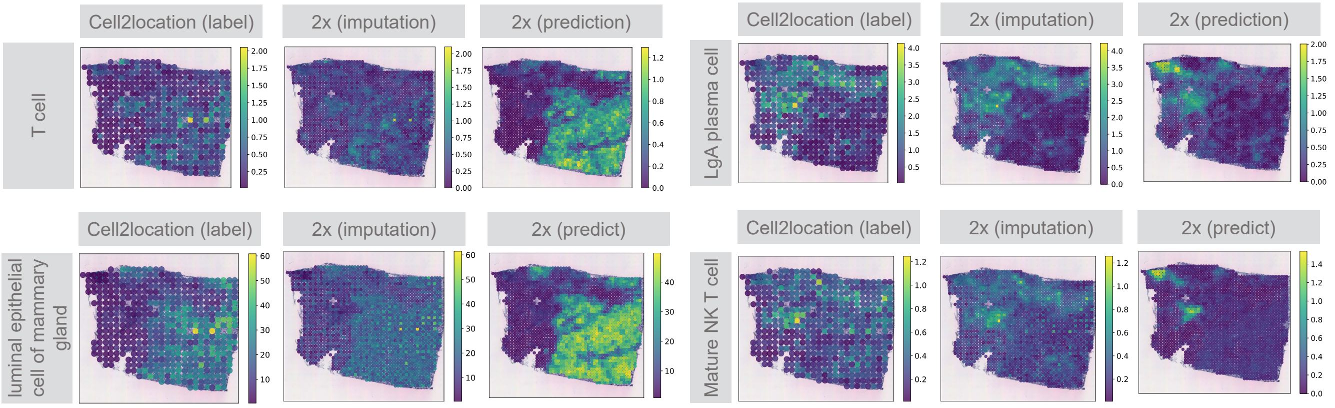

Figure S43. Hist2Cell provides super-resolved spatial cell abundances with more accurate alignment to tumor/normal annotation than STNet, related to Figure 6

Visualizations comparing low-resolution ground truth cell abundance with both imputed and directly predicted high-resolution results by Hist2Cell. Both approaches provide a more detailed mapping in concordance with the tumor/normal annotation shown in Figure 4C. Color intensity represents the cell abundance.

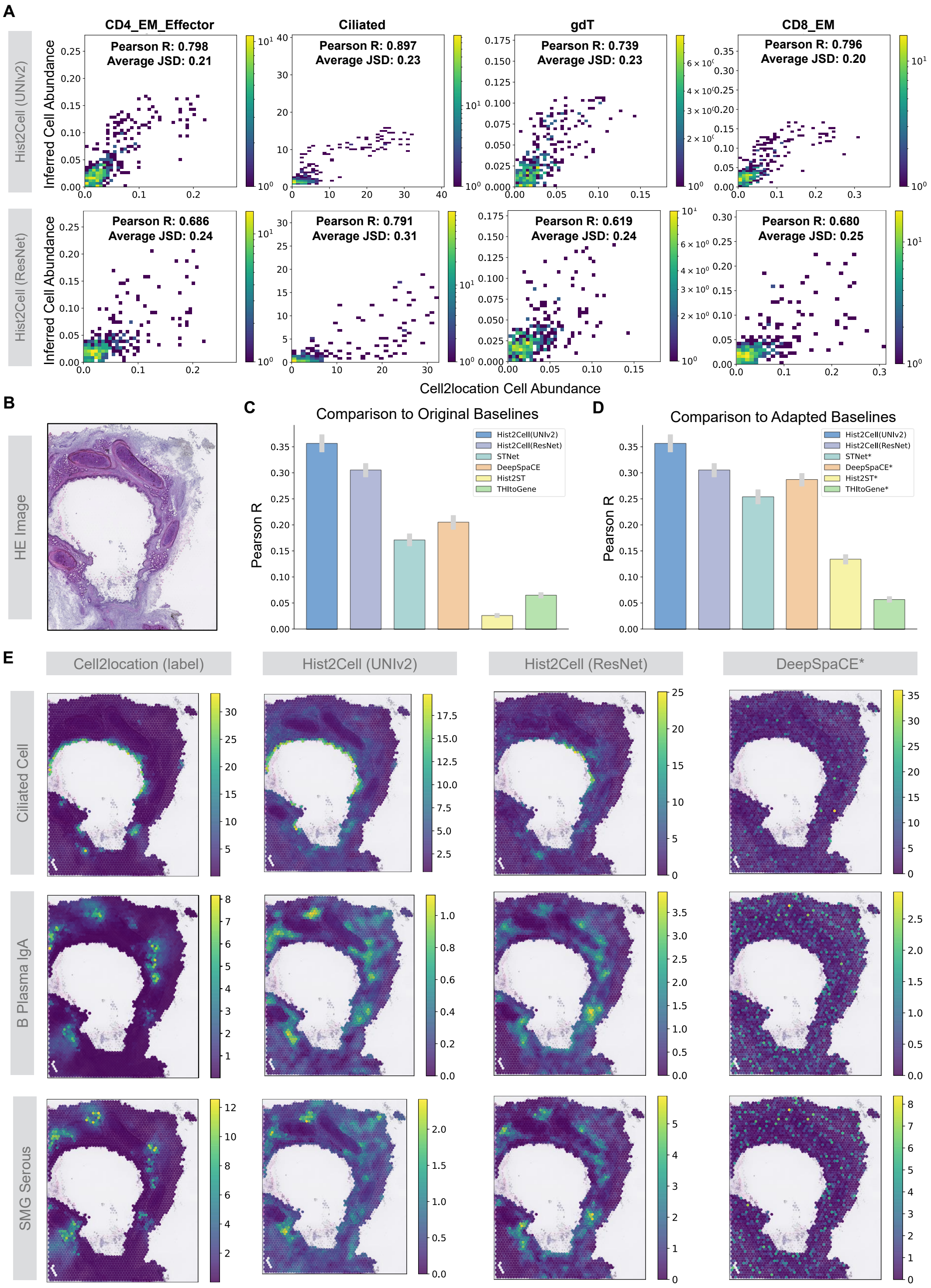

Figure S44. Performance comparison on human lung dataset, related to Discussion

Hist2Cell(UNIv2) is the enhanced version of our framework using the pathology foundation model as image encoder, while Hist2Cell(ResNet) uses ImageNet pretrained ResNet as the image encoder.

(A) 2D histogram plots showcasing the concordance of cell abundance between ground truth (x-axis) and model's prediction (y-axis) across all testing spots in the healthy human lung slide. Color denotes 2D histogram counts. Pearson's R denotes Pearson's correlation coefficient, and JSD denotes Jensen–Shannon divergence. Color intensity represents the spot frequency.

(B) Example H&E image for slide (ID) 9258464.

(C, D) Histogram depicting the average Pearson's R values for cell abundance prediction in the leave-one-donor-out cross-validation experiment conducted on the healthy human lung dataset. * means adapting the two-stage ST prediction baseline to our one-stage prediction strategy. Error bars represent standard error across the cross-validation folds.

(E) Related to (B), the visualizations comparing the key spatial cell abundances as determined by ground truth, Hist2Cell, and DeepSpaCE* predictions. Hist2Cell shows fewer false positives than DeepSpaCE*(the best-performing adapted ST prediction baseline). Color intensity represents the cell abundance.

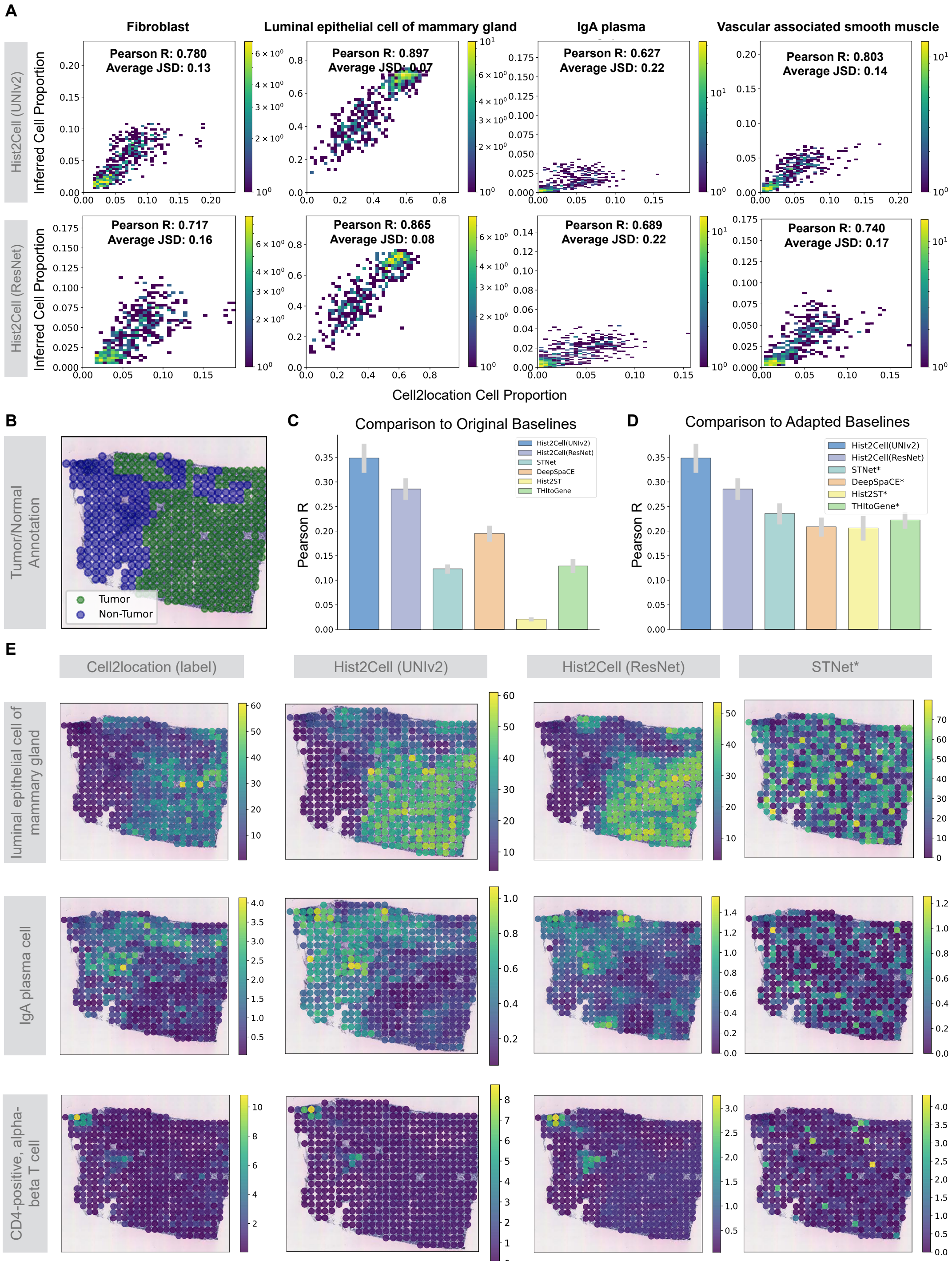

Figure S45. Performance comparison on external unseen breast cancer dataset, related to Discussion

Hist2Cell(UNIv2) is the enhanced version of our framework using the pathology foundation model as image encoder, while Hist2Cell(ResNet) uses ImageNet pretrained ResNet as the image encoder.

(A) 2D histogram plots showcasing the concordance of cell abundance between ground truth (x-axis) and model's prediction (y-axis) across all testing spots in the breast cancer slide. Color denotes 2D histogram counts. Pearson's R denotes Pearson's correlation coefficient, and JSD denotes Jensen–Shannon divergence. Color intensity represents the spot frequency.

(B) Expert manual tumor/normal annotation for slide (ID) 23508D2.

(C, D) Histogram representing the average Pearson's R values for cell abundance prediction in the external breast cancer dataset. * means adapting the two-stage ST prediction baseline to our one-stage prediction strategy. Error bars represent standard error across the cross-validation folds.
(E) Related to (B), the visualizations comparing the key spatial cell abundances as determined by ground truth, Hist2Cell, and STNet* predictions. Hist2Cell shows fewer false positives than STNet*(the best-performing adapted ST prediction baseline). Color intensity represents the cell abundance.

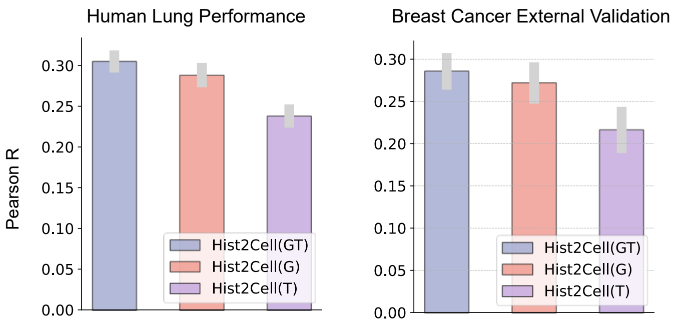

Figure S46. Ablation study on the contribution of GNN and Transformer components, related STAR Methods

Histogram depicting the average Pearson's R values for cell abundance prediction for human lung dataset and external breast cancer dataset. Error bars represent standard error across the cross-validation folds. "GT" represents the complete Hist2Cell, "G" represents removing the Transformer part of Hist2Cell, and "T" represents removing the GNN part of Hist2Cell. Results show that both the GNN part and the Transformer part contribute to the performance increase of Hist2Cell, validating the efficacy of utilizing both local and global information within the tissue.

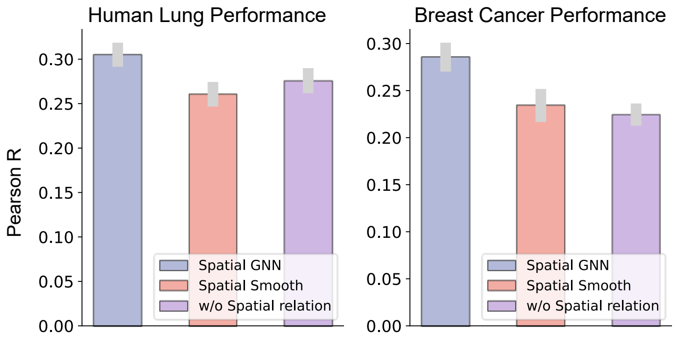

Figure S47. Impact of spatial relation modeling on prediction performance, related STAR Methods

Histogram depicting the average Pearson's R values for cell abundance prediction for human lung dataset and breast cancer datasets. Error bars represent standard error across the cross-validation folds. We compare three different settings: using GNN, using spatial smoothing from STNet and removing the spatial relation modeling in Hist2Cell.

SUPPLEMENTAL TABLES

Table S1. Survival performance comparison, related to Figure 5

Model	Pre-training Slide No.	C-index(%)			
		BRCA-HER2+	BRCA-TNBC	LUSC	Average
HIPT[42]	10,678	57.82	71.57	70.58	66.65
UNI[44]	100,426	64.05	74.35	**71.75**	70.05
Hist2Cell	114 (BRCA) / 34 (LUSC)	**67.89**	**74.43**	71.59	**71.30**
Bulk RNA-seq	N/A	70.75	70.60	72.05	71.13

Comparison average C-indices of the cox regression models predicting survival of three different cancer subtypes: LUSC, BRCA-TNBC, and BRCA-HER2+. C-indices were calculated from the test sets of a 10-fold Cross-Validation experiment. The Cox regression models used the predicted cell abundances of Hist2Cell and the image feature extracted by HIPT/UNI, and the bulk-RNA seq of the patients as inputs, respectively. Top performance in the histology image-based models is shown in **bold**.

Table S2. Metadata of HEST1K samples Part 1, related to STAR Methods

ID	Organ	Technique	ID	Organ	Technique
SPA0	Breast	Spatial Transcriptomics	SPA140	Breast	Spatial Transcriptomics
SPA1	Breast	Spatial Transcriptomics	SPA141	Breast	Spatial Transcriptomics
SPA100	Breast	Spatial Transcriptomics	SPA142	Breast	Spatial Transcriptomics
SPA101	Breast	Spatial Transcriptomics	SPA143	Breast	Spatial Transcriptomics
SPA102	Breast	Spatial Transcriptomics	SPA144	Breast	Spatial Transcriptomics
SPA103	Breast	Spatial Transcriptomics	SPA145	Breast	Spatial Transcriptomics
SPA104	Breast	Spatial Transcriptomics	SPA146	Breast	Spatial Transcriptomics
SPA105	Breast	Spatial Transcriptomics	SPA147	Breast	Spatial Transcriptomics
SPA106	Breast	Spatial Transcriptomics	SPA148	Breast	Spatial Transcriptomics
SPA107	Breast	Spatial Transcriptomics	SPA149	Breast	Spatial Transcriptomics
SPA108	Breast	Spatial Transcriptomics	SPA150	Breast	Spatial Transcriptomics
SPA109	Breast	Spatial Transcriptomics	SPA151	Breast	Spatial Transcriptomics
SPA110	Breast	Spatial Transcriptomics	SPA152	Breast	Spatial Transcriptomics
SPA111	Breast	Spatial Transcriptomics	SPA153	Breast	Spatial Transcriptomics
SPA112	Breast	Spatial Transcriptomics	SPA154	Breast	Spatial Transcriptomics
SPA113	Breast	Spatial Transcriptomics	SPA2	Breast	Spatial Transcriptomics
SPA114	Breast	Spatial Transcriptomics	SPA3	Breast	Spatial Transcriptomics
SPA115	Breast	Spatial Transcriptomics	SPA51	Breast	Spatial Transcriptomics
SPA116	Breast	Spatial Transcriptomics	SPA52	Breast	Spatial Transcriptomics
SPA117	Breast	Spatial Transcriptomics	SPA53	Breast	Spatial Transcriptomics
SPA118	Breast	Spatial Transcriptomics	SPA54	Breast	Spatial Transcriptomics
SPA119	Breast	Spatial Transcriptomics	SPA55	Breast	Spatial Transcriptomics
SPA120	Breast	Spatial Transcriptomics	SPA56	Breast	Spatial Transcriptomics
SPA121	Breast	Spatial Transcriptomics	SPA57	Breast	Spatial Transcriptomics
SPA122	Breast	Spatial Transcriptomics	SPA58	Breast	Spatial Transcriptomics
SPA123	Breast	Spatial Transcriptomics	SPA59	Breast	Spatial Transcriptomics
SPA124	Breast	Spatial Transcriptomics	SPA60	Breast	Spatial Transcriptomics
SPA125	Breast	Spatial Transcriptomics	SPA61	Breast	Spatial Transcriptomics
SPA126	Breast	Spatial Transcriptomics	SPA62	Breast	Spatial Transcriptomics
SPA127	Breast	Spatial Transcriptomics	SPA63	Breast	Spatial Transcriptomics
SPA128	Breast	Spatial Transcriptomics	SPA64	Breast	Spatial Transcriptomics
SPA129	Breast	Spatial Transcriptomics	SPA65	Breast	Spatial Transcriptomics
SPA130	Breast	Spatial Transcriptomics	SPA66	Breast	Spatial Transcriptomics
SPA131	Breast	Spatial Transcriptomics	SPA67	Breast	Spatial Transcriptomics
SPA132	Breast	Spatial Transcriptomics	SPA68	Breast	Spatial Transcriptomics
SPA133	Breast	Spatial Transcriptomics	SPA69	Breast	Spatial Transcriptomics
SPA134	Breast	Spatial Transcriptomics	SPA70	Breast	Spatial Transcriptomics
SPA135	Breast	Spatial Transcriptomics	SPA71	Breast	Spatial Transcriptomics
SPA136	Breast	Spatial Transcriptomics	SPA72	Breast	Spatial Transcriptomics
SPA137	Breast	Spatial Transcriptomics	SPA73	Breast	Spatial Transcriptomics
SPA138	Breast	Spatial Transcriptomics	SPA74	Breast	Spatial Transcriptomics
SPA139	Breast	Spatial Transcriptomics	SPA75	Breast	Spatial Transcriptomics

The slide IDs and metadata of the HEST1K dataset samples (Part 1/2).

Table S3. Metadata of HEST1K samples Part 2, related to STAR Methods

ID	Organ	Technique	ID	Organ	Technique
SPA76	Breast	Spatial Transcriptomics	MISC25	Lung	Visium
SPA77	Breast	Spatial Transcriptomics	MISC26	Lung	Visium
SPA78	Breast	Spatial Transcriptomics	MISC27	Lung	Visium
SPA79	Breast	Spatial Transcriptomics	MISC28	Lung	Visium
SPA80	Breast	Spatial Transcriptomics	MISC29	Lung	Visium
SPA81	Breast	Spatial Transcriptomics	MISC30	Lung	Visium
SPA82	Breast	Spatial Transcriptomics	MISC31	Lung	Visium
SPA83	Breast	Spatial Transcriptomics	MISC32	Lung	Visium
SPA84	Breast	Spatial Transcriptomics	TENX62	Lung	Visium
SPA85	Breast	Spatial Transcriptomics	NCBI469	Skin	Visium
SPA86	Breast	Spatial Transcriptomics	NCBI470	Skin	Visium
SPA87	Breast	Spatial Transcriptomics	NCBI471	Skin	Visium
SPA88	Breast	Spatial Transcriptomics	NCBI472	Skin	Visium
SPA89	Breast	Spatial Transcriptomics	NCBI473	Skin	Visium
SPA90	Breast	Spatial Transcriptomics	NCBI474	Skin	Visium
SPA91	Breast	Spatial Transcriptomics	NCBI475	Skin	Visium
SPA92	Breast	Spatial Transcriptomics	NCBI476	Skin	Visium
SPA93	Breast	Spatial Transcriptomics	NCBI477	Skin	Visium
SPA94	Breast	Spatial Transcriptomics	NCBI478	Skin	Visium
SPA95	Breast	Spatial Transcriptomics	NCBI479	Skin	Visium
SPA96	Breast	Spatial Transcriptomics	NCBI480	Skin	Visium
SPA97	Breast	Spatial Transcriptomics	NCBI481	Skin	Visium
SPA98	Breast	Spatial Transcriptomics	NCBI482	Skin	Visium
SPA99	Breast	Spatial Transcriptomics	NCBI483	Skin	Visium
MISC13	Lung	Visium	NCBI484	Skin	Visium
MISC14	Lung	Visium	NCBI485	Skin	Visium
MISC15	Lung	Visium	NCBI486	Skin	Visium
MISC16	Lung	Visium	NCBI487	Skin	Visium
MISC17	Lung	Visium	NCBI488	Skin	Visium
MISC18	Lung	Visium	NCBI489	Skin	Visium
MISC19	Lung	Visium	NCBI490	Skin	Visium
MISC20	Lung	Visium	NCBI491	Skin	Visium
MISC21	Lung	Visium	NCBI492	Skin	Visium
MISC22	Lung	Visium	NCBI493	Skin	Visium
MISC23	Lung	Visium	NCBI494	Skin	Visium
MISC24	Lung	Visium	NCBI495	Skin	Visium
NCBI496	Skin	Visium	NCBI509	Skin	Visium
NCBI497	Skin	Visium	NCBI510	Skin	Visium
NCBI498	Skin	Visium	NCBI515	Skin	Visium
NCBI503	Skin	Visium	NCBI516	Skin	Visium
NCBI504	Skin	Visium	NCBI517	Skin	Visium
NCBI505	Skin	Visium	NCBI518	Skin	Visium
NCBI506	Skin	Visium	NCBI519	Skin	Visium
NCBI507	Skin	Visium	NCBI520	Skin	Visium
NCBI508	Skin	Visium	NCBI521	Skin	Visium
NCBI522	Skin	Visium			

The slide IDs and metadata of the HEST1K dataset samples (Part 2/2).
